# Metabolically-healthy obesity is associated with higher prevalence of colorectal adenoma

**DOI:** 10.1371/journal.pone.0179480

**Published:** 2017-06-21

**Authors:** Dong Hyun Sinn, Yang Won Min, Hee Jung Son, Poong-Lyul Rhee, Seung Woon Paik, Sung Noh Hong, Geum-Youn Gwak

**Affiliations:** 1 Department of Medicine, Samsung Medical Center, Sungkyunkwan University School of Medicine, Seoul, South Korea; 2 Center for Health Promotion, Samsung Medical Center, Seoul, South Korea; University Hospital Llandough, UNITED KINGDOM

## Abstract

**Background:**

The risk of colorectal adenoma (CRA), an important precursor of colorectal cancer, is largely unknown among obese individuals without obesity-related metabolic abnormalities, a condition described as metabolically-healthy obese (MHO). The aim of this study was to examine the association among metabolic status, the different categories of body mass index (BMI), and CRA in a large cohort of adults.

**Methods:**

We analyzed the association among metabolic status, BMI categories and CRA in asymptomatic adults who underwent a first-time colonoscopy as part of the comprehensive health check-up program at the Health Promotion Center of Samsung Medical Center, from January 2005 to December 2012. Being metabolically healthy was defined as lacking any metabolic syndrome components and having a homeostasis model assessment of insulin resistance <2.5.

**Results:**

The prevalence of “any,” “multiple,” and “high-risk” CRA was 25.6%, 8.3%, and 4.4% among 9,182 metabolically-healthy participants, and 35.9%, 12.5%, and 7.0% among 17,407 metabolically-unhealthy participants, respectively. Increased BMI showed a significant dose-dependent relationship with the prevalence of “any,” “multiple,” and “high-risk” CRA, in both metabolically-healthy and unhealthy participants. In multivariable-adjusted models that accounted for potential confounders including age, sex, smoking, alcohol, first-degree family history of colorectal cancer, and aspirin use, the odds ratio (OR) for any CRA comparing MHO with metabolically-healthy normal-weight (MHNW) participants was 1.25 (95% confidence interval (CI), 1.09–1.43). Further adjustment for metabolic components associated with obesity did not significantly change the association. Similarly, the ORs for multiple CRAs and high risk CRA were higher in MHO participants than MHNW participants [ORs (95% CI), 1.63 (1.31–2.04) and 1.53 (1.14–2.04), respectively].

**Conclusions:**

The MHO phenotype was closely associated with higher prevalence of CRA, including high-risk adenoma. This finding supports the conclusion that MHO increases the risk of colorectal cancer.

## Introduction

Colorectal cancer (CRC) is a major public health problem, currently being the third most-commonly diagnosed cancer, and the fourth highest cause of cancer mortality worldwide [1]. Moreover, the global burden is expected to increase further due to the aging of the population and the spread of the westernized lifestyle.

In addition to several genetic factors, many studies have attributed the increased risk of CRC to “environmental factors,” living the “westernized lifestyle” [2, 3], which encompasses obesity, sedentary behavior, and a high-calorie, fat-rich, fiber-deficient diet. Epidemiological evidence, including meta-analysis comprised of approximately 9,000,000 participants from different countries, strongly support a positive association between obesity and CRC [4]. Several mechanisms linking obesity to CRC have been proposed: obesity-related insulin resistance, hyperinsulinemia, sustained hyperglycemia, and a hyperinsulinemia-related increase of insulin-like growth factor-1 [5]. A number of studies have also demonstrated the positive association between body mass index (BMI) and colorectal adenoma (CRA), an important precursor to the subsequent development of CRC [6]. An increased understanding of CRA and its related conditions can be useful in determining the benefits of early CRC screening.

A subset of the obese population without obesity-related metabolic abnormalities [7, 8], often referred to as metabolically-healthy obese (MHO), are relatively insulin sensitive [9]. The health implications of MHO are controversial [10–12]. Moreover, the risk of CRC or CRA among MHO individuals is largely unknown. To date, only one study is available for the association between MHO and CRA, which is limited by the young age of the study population (mean age of 39.7 years) and low detection rate of CRA (9.3% and 1.4% for low and high risk adenoma, respectively) [13]. Therefore, we examined the association among metabolic status, BMI categories, and CRA in a large cohort of adults undergoing a first-time colonoscopy.

## Materials and methods

### Study design, setting, participants

The study population was comprised of adult men and women aged 40 years or older who underwent a comprehensive health check-up program at the Health Promotion Center of Samsung Medical Center in Seoul, South Korea, from January 2005 to December 2012. Study subjects were restricted to participants who underwent a complete colonoscopy for the first time, with adequate bowel preparation, and had no previous history of cancer or inflammatory bowel disease (*n* = 37,004). We then excluded 10,415 participants with missing information on blood pressure, fasting blood glucose, lipid profiles, height or weight, and/or homeostasis model assessment of insulin resistance (HOMA-IR). The final sample size was 26,589 participants (Table 1). The Institutional Review Board of the Samsung Medical Center approved this study and waived the requirement for informed consent as we used only de-identified data routinely collected during health screening visits.

**Table 1:** Flow diagram of study participants.

Screening (n = 37,004)	≥40 years old who underwent first time, complete colonoscopy with adequate bowel preparation, no previous history of cancer or inflammatory bowel disease at Health Promotion Center of the Samsung Medical Center between January 2005 and December 2012
Participants with missing data^a^ (n = 10,415)	Blood pressure (n = 19)
	Fasting blood glucose (n = 3)
	Lipid profiles (n = 196)
	Height or weight (n = 18)
	HOMA-IR (n = 10,402)
Final study sample (n = 26,589)	

^a^ Participants in the screening program could have > 1 criterion that made them ineligible for the study.

HOMA-IR, homeostasis model assessment of insulin resistance.

### Measurement of variables

During a comprehensive health check-up, demographic characteristics, smoking status, alcohol consumption, medical history, family history (including first-degree family history of CRC), and use of medications were collected through standardized, self-administered questionnaires (S1 and S2 Questionnaires). Smoking status was categorized into “never,” “former,” “current smoker,” or missing. Alcohol consumption was categorized as “no” or “yes,” according to the current drinking status. Height, weight and sitting blood pressure were measured by trained nurses. BMI was calculated as weight in kilograms, divided by height in meters, squared. BMI was classified according to Asian-specific criteria [14]: underweight, BMI <18.5 kg/m^2^; normal weight, BMI of 18.5 to 23 kg/m^2^; overweight, BMI of 23 to 25 kg/m^2^; and obese, BMI ≥25 kg/m^2^.

Laboratory evaluation including serum glucose, lipid profile, and blood insulin was measured by standard methods after overnight fasting. Insulin resistance was assessed with the HOMA-IR, according to the following equation: fasting blood insulin (μU/ml) × fasting serum glucose (mmol/l)/22.5. The Department of Laboratory Medicine and Genetics at Samsung Medical Center has participated in several proficiency testing programs operated by the Korean Association of Quality Assurance for Clinical Laboratory, the Asian Network of Clinical Laboratory Standardization and Harmonization, and the College of American Pathologists.

### Definition of metabolic status

Participants were defined as metabolically-unhealthy for those who had any of the following metabolic abnormalities [13]: 1) fasting blood glucose ≥100 mg/dl or current use of blood glucose-lowering agents; 2) blood pressure ≥130/85 mmHg or current use of blood pressure-lowering agents; 3) triglyceride levels ≥150 mg/dl or current use of lipid-lowering agents; 4) high-density lipoprotein cholesterol (HDL-C) <40 mg/dl in men or <50 mg/dl in women; or 5) HOMA-IR ≥2.5. Those without any of the metabolic abnormalities were defined as metabolically-healthy. Waist circumference was not used to define metabolic status.

### Colonoscopy

All colonoscopies were performed by board-certified endoscopists, after bowel preparation with polyethylene glycol solutions. A complete examination was defined as one that reached the cecum, with a picture of the ileocecal valve obtained following adequate bowel preparation. All polypoid lesions were biopsied or removed with records of the location, size, number and appearance.

All the CRAs were histologically evaluated and classified according to the World Health Organization standards [15]. High-risk adenoma was defined as more than three adenomas and/or adenoma with villous histology, high-grade dysplasia, or size >10 mm.

### Statistical analysis

The difference between groups was tested using the t-test, Chi-square test, or Kruskal-Wallis test, as appropriate. The difference in the prevalence of any adenoma, multiple adenomas, and high-risk adenoma for baseline BMI categories was assessed using logistic regression analysis. We used three models with increasing levels of adjustment to account for potential confounders and mediators. Model 1 was adjusted for age and sex. Model 2 was further adjusted for smoking, alcohol, first-degree family history of colon cancer, and aspirin use. Model 3 was additionally adjusted for fasting blood glucose, systolic blood pressure, triglyceride, HDL-C, low-density lipoprotein cholesterol (LDL-C), and HOMA-IR, to account for any possible mediation of the association between MHO and CRA by metabolic components. All reported *P* values were two-sided and the significance level was set at 0.05. These analyses were conducted using SAS version 9.4 (SAS Institute, Cary, NC).

## Results

Overall, a total of 26,589 participants (mean age = 51.9 years, range = 40–89 years; male = 14,703 (55.3%); mean BMI = 23.8 kg/m^2^, range = 14.0–45.0 kg/m^2^) were analyzed. Baseline characteristics between metabolically-healthy participants (n = 9,182) and metabolically-unhealthy participants (n = 17,407) were significantly different (Table 2). Metabolically-unhealthy participants were more likely to be older, male, obese, former or current smokers, and current drinkers. The prevalence of any, multiple, and high-risk CRA was higher for metabolically-unhealthy participants. BMI (per unit) was associated with higher prevalence of any, multiple and high-risk CRA (S1 Table). This association was apparent both in metabolically-healthy and metabolically-unhealthy participants.

**Table 2:** Comparison of baseline characteristics by metabolic status.

	Metabolically-healthy (n = 9,182)	Metabolically-unhealthy (n = 17,407)	P value
Age, years	50.0 ± 6.2	52.9 ± 7.4	< 0.001
Male	3,630 (39.5)	11,073 (63.6)	< 0.001
BMI, kg/m^2^	22.3 ± 2.4	24.6 ± 2.8	< 0.001
Smoking			< 0.001
Never	5,725 (62.4)	7,734 (44.4)	
Former	1,080 (11.8)	3,186 (18.3)	
Current	1,370 (14.9)	3,890 (22.3)	
Missing	1,007 (11.0)	2,597 (14.9)	
Alcohol			< 0.001
No	3,315 (36.1)	5,230 (30.0)	
Yes	5,390 (58.7)	11,187 (64.3)	
Missing	477 (5.2)	990 (5.7)	
First-degree family history of CRC	205 (2.2)	339 (1.9)	0.11
Aspirin use	223 (2.4)	2,060 (11.8)	< 0.001
Systolic blood pressure, mmHg	109 (101–116)	122 (111–135)	< 0.001
Fasting blood glucose, mg/dL	87 (82–91)	96 (88–105)	< 0.001
Triglycerides, mg/dL	77 (59–100)	128 (89–178)	< 0.001
LDL-C, mg/dL	119 (101–139)	126 (105–147)	< 0.001
HDL-C, mg/dL	61 (53–70)	48 (41–57)	< 0.001
HOMA-IR	1.17 (0.79–1.59)	1.92 (1.33–2.71)	< 0.001
Any adenoma	2,351 (25.6)	6,244 (35.9)	< 0.001
Multiple adenomas	761 (8.3)	2,182 (12.5)	< 0.001
High-risk adenoma^a^	401 (4.4)	1,216 (7.0)	< 0.001
Invasive cancer	11 (0.1)	34 (0.2)	0.15

Values in table are expressed as number (percentage), mean ± standard deviation or median (quartile). BMI, body mass index; CRC, colorectal cancer; LDL-C, low-density lipoprotein cholesterol; HDL-C, high-density lipoprotein cholesterol; HOMA-IR, homeostasis model assessment of insulin resistance.

^a^ High-risk adenoma was defined as more than three adenomas and/or adenoma with villous histology, high-grade dysplasia, or size >10 mm.

When participants were categorized according to the BMI, participants belonging to higher BMI categories were more likely to be older, male, former/current smokers, and current alcohol drinkers, and to have higher levels of systolic blood pressure, fasting blood glucose, triglycerides, LDL-C, and HOMA-IR, and lower levels of HDL-C than participants belonging to lower BMI categories, in both metabolically-healthy and unhealthy participants (Table 3).

**Table 3:** Comparison of baseline characteristics by body mass index category and metabolic status.

	Metabolically-healthy participants					Metabolically-unhealthy participants				
	Underweight (n = 373)	Normal (n = 5,199)	Overweight (n = 2,195)	Obese (n = 1,415)	P value	Underweight (n = 144)	Normal (n = 4,838)	Overweight (n = 5,052)	Obese (n = 7,373)	P value
Age, years	48.9 ± 7.0	49.5 ± 6.0	50.8 ± 6.4	50.6 ± 6.3	< 0.001	51.0 ± 7.1	53.0 ± 7.3	53.4 ± 7.3	52.7 ± 7.4	< 0.001
Male	52 (13.9)	1,421 (27.3)	1191 (54.3)	966 (68.3)	< 0.001	35 (24.3)	2,172 (44.9)	3,357 (66.4)	5,509 (74.7)	< 0.001
BMI, kg/m^2^	17.6 ± 0.6	21.0 ± 1.1	23.8 ± 0.5	26.4 ± 1.3	< 0.001	17.6 ± 0.7	21.5 ± 1.0	24.0 ± 0.5	27.1 ± 1.9	< 0.001
Smoking					< 0.001					< 0.001
Never	283 (75.9)	3,702 (71.2)	1,119 (51.0)	621 (43.9)		105 (72.9)	2,754 (56.9)	2,163 (42.8)	2,712 (36.8)	
Former	16 (4.3)	409 (7.9)	368 (16.8)	287 (20.3)		5 (3.5)	617 (12.8)	992 (19.6)	1,572 (21.3)	
Current	40 (10.7)	612 (11.8)	421 (19.2)	297 (21.0)		23 (16.0)	848 (17.5)	1,146 (22.7)	1,873 (25.4)	
Missing	34 (9.1)	476 (9.2)	287 (13.1)	210 (14.8)		11 (7.6)	619 (12.8)	751 (14.9)	1,216 (16.5)	
Alcohol					< 0.001					< 0.001
No	196 (52.5)	2,148 (41.3)	636 (29.0)	335 (23.7)		86 (59.7)	1,896 (39.2)	1,455 (28.8)	1,793 (24.3)	
Yes	155 (41.6)	2,799 (53.8)	1,442 (65.7)	994 (70.2)		56 (38.9)	2,674 (55.3)	3,306 (65.4)	5,151 (69.9)	
Missing	22 (5.9)	252 (4.8)	117 (5.3)	86 (6.1)		2 (1.4)	268 (5.5)	291 (5.8)	429 (5.8)	
FHx of CRC	7 (1.9)	123 (2.4)	48 (2.2)	27 (1.9)	0.71	4 (2.8)	103 (2.1)	104 (2.1)	128 (1.7)	0.71
Aspirin use	7 (1.9)	97 (1.9)	58 (2.6)	61 (4.3)	< 0.001	11 (7.6)	442 (9.1)	626 (12.4)	981 (13.3)	< 0.001
SBP, mmHg	105 (96–111)	107 (100–115)	110 (103–118)	113 (105–119)	< 0.001	114 (101–132)	119 (108–133)	121 (111–134)	124 (114–136)	< 0.001
FBG, mg/dL	85 (80–90)	86 (81–91)	87 (83–92)	88 (83–93)	< 0.001	93 (84–102)	93 (87–103)	95 (88–104)	97 (89–107)	< 0.001
TG, mg/dL	64 (52–83)	72 (56–92)	83 (64–107)	89 (69–114)	< 0.001	79 (63–111)	104 (74–154)	128 (91–176)	143 (102–196)	< 0.001
LDL-C, mg/dL	108 (93–125)	116 (98–136)	124 (106–145)	127 (108–145)	< 0.001	104 (85–126)	122 (102–142)	126 (106–147)	128 (107–149)	< 0.001
HDL-C, mg/dL	68 (60–78)	63 (56–73)	58 (51–66)	54 (48–62)	< 0.001	63 (50–75)	51 (44–62)	48 (41–57)	46 (39–54)	< 0.001
HOMA-IR	0.9 (0.5–1.3)	1.1 (0.7–1.5)	1.3 (0.9–1.7)	1.5 (1.1–1.9)	< 0.001	1.1 (0.7–1.7)	1.5 (1.1–2.2)1	1.8 (1.3–2.5)	2.3 (1.7–3.2)	< 0.001
Any adenoma	75 (20.1)	1,187 (22.8)	617 (28.1)	472 (33.4)	< 0.001	32 (22.2)	1,452 (30.0)	1,797 (35.6)	2,963 (40.2)	< 0.001
Multiple adenomas	14 (3.8)	327 (6.3)	231 (10.5)	189 (13.4)	< 0.001	10 (6.9)	459 (9.5)	621 (12.3)	1,092 (14.8)	< 0.001
High-risk adenoma	11 (2.9)	184 (3.5)	108 (4.9)	98 (6.9)	< 0.001	5 (3.5)	260 (5.4)	330 (6.5)	521 (8.4)	< 0.001
Invasive cancer	0	5 (0.1)	5 (0.2)	1 (0.1)	0.37	0	10 (0.2)	7 (0.1)	17 (0.2)	0.37

Values in table are expressed as number (percentage), mean ± standard deviation or median (quartile). BMI, body mass index; FHx of CRC, First degree family history of colorectal cancer; SBP, systolic blood pressure; FBG, fasting blood glucose; TG, triglyceride; LDL-C, low-density lipoprotein cholesterol; HDL-C, high-density lipoprotein cholesterol; HOMA-IR, homeostasis model assessment of insulin resistance.

In multivariable-adjusted models that accounted for potential confounders including age, sex, smoking, alcohol, first-degree family history of colorectal cancer, and aspirin use, the odds ratio (OR) for any CRA comparing MHO with metabolically-healthy normal-weight (MHNW) participants was 1.25 (95% confidence interval (CI), 1.09–1.43) (Table 4). To explore whether the increased prevalence in any CRA observed in MHO participants was mediated by metabolic risk factors associated with obesity, we performed additional analyses adjusted for metabolic components. Adjustments for fasting blood glucose, systolic blood pressure, triglyceride levels, HDL-C, LDL-C, and HOMA-IR did not virtually change the association.

**Table 4:** Odd ratios of colorectal adenoma by body mass index category in metabolically-healthy participants (n = 9,182).

	Underweight	Normal	Overweight	Obese
Any adenoma				
Unadjusted	0.85 (0.65–1.10)	reference	1.32 (1.18–1.48)	1.69 (1.48–1.92)
Adjusted				
Model 1	0.95 (0.73–1.24)	reference	1.03 (0.92–1.17)	1.23 (1.07–1.41)
Model 2	0.94 (0.72–1.23)	reference	1.03 (0.91–1.16)	1.25 (1.09–1.43)
Model 3	0.95 (0.73–1.25)	reference	1.02 (0.90–1.16)	1.24 (1.07–1.43)
Multiple adenomas^a^				
Unadjusted	0.57 (0.33–0.99)	reference	1.79 (1.50–2.14)	2.45 (2.02–2.91)
Adjusted				
Model 1	0.64 (0.36–1.14)	reference	1.26 (1.04–1.52)	1.60 (1.30–1.96)
Model 2	0.63 (0.35–1.12)	reference	1.26 (1.04–1.53)	1.65 (1.34–2.03)
Model 3	0.64 (0.36–1.15)	reference	1.25 (1.03–1.52)	1.63 (1.31–2.04)
High-risk adenoma^a^				
Unadjusted	0.80 (0.43–1.49)	reference	1.49 (1.16–1.90)	2.26 (1.75–2.92)
Adjusted				
Model 1	0.90 (0.47–1.72)	reference	1.05 (0.81–1.36)	1.47 (1.12–1.93)
Model 2	0.90 (0.47–1.72)	reference	1.05 (0.81–1.35)	1.53 (1.16–2.01)
Model 3	0.90 (0.47–1.73)	reference	1.04 (0.80–1.36)	1.53 (1.14–2.04)

Values in parenthesis are 95% confidence intervals.

^a^ Compared to individuals without adenoma. Model 1: Adjusted for age and sex. Model 2: Further adjusted for smoking, alcohol, first-degree family history of colorectal cancer, and aspirin use. Model 3: Further adjusted for fasting blood glucose, systolic blood pressure, triglyceride, high-density lipoprotein cholesterol, low-density lipoprotein cholesterol, and HOMA-IR.

In multivariable-adjusted models that accounted for potential confounders and metabolic components, the ORs for multiple CRAs were higher in MHO participants than in MHNW participants [ORs (95% CI), 1.63 (1.31–2.04)] (Table 4). Also, the OR for high-risk CRA was higher in MHO participants than in MHNW participants [OR (95% CI), 1.53 (1.14–2.04)].

The prevalence of any, multiple and high-risk adenoma was higher in men than women (34.8% vs. 19.6% for any adenoma; 13.7% vs. 4.7% for multiple adenoma; 7.1% vs. 2.6% for high-risk adenoma, respectively; p < 0.001 for all). In sub-group analysis according to sex, MHO participants showed higher prevalence and multivariable-adjusted ORs of any, multiple, and high-risk adenoma compared to MHNW participants regardless of sex, although the difference did not reach statistical significance for any adenoma and high risk adenoma in men (S2 Table).

When MHO participants were used as a reference, the un-adjusted ORs of any, multiple and high-risk adenoma were lower in MHNW participants and metabolically-unhealthy normal-weight (MUNW) participants, similar in metabolically-unhealthy overweight (MUOW) participants, and higher in metabolically-unhealthy obese (MUO) participants (Table 5). In the multivariable-adjusted model 2, the ORs of any adenoma were significantly lower in MHNW and MUNW participants, similar in MUOW participants and higher in MUO participants. The ORs of multiple and high-risk adenoma were lower in MHNW, MUNW and MUOW participants and were similar in MUO participants.

**Table 5:** Comparison of odd ratios of colorectal adenoma according to body mass index category and metabolic status.

	Any adenoma	Multiple adenoma^a^	High-risk adenoma^a^
Unadjusted			
Metabolically-healthy, obesity	Reference	Reference	Reference
Metabolically-healthy, normal-weight	0.59 (0.52–0.67)	0.40 (0.33–0.49)	0.44 (0.34–0.57)
Metabolically-unhealthy, normal-weight	0.85 (0.75–0.97)	0.67 (0.56–0.81)	0.73 (0.57–0.94)
Metabolically-unhealthy, overweight	1.10 (0.97–1.24)	0.95 (0.79–1.13)	0.97 (0.77–1.23)
Metabolically-unhealthy, obesity	1.34 (1.19–1.51)	1.23 (1.04–1.46)	1.35 (1.08–1.69)
Model 1			
Metabolically-healthy, obesity	Reference	Reference	Reference
Metabolically-healthy, normal-weight	0.82 (0.72–0.94)	0.65 (0.58–0.80)	0.72 (0.55–0.94)
Metabolically-unhealthy, normal-weight	0.89 (0.78–1.01)	0.69 (0.57–0.84)	0.74 (0.58–0.96)
Metabolically-unhealthy, overweight	0.97 (0.86–1.11)	0.77 (0.64–0.93)	0.77 (0.60–0.98)
Metabolically-unhealthy, obesity	1.17 (1.04–1.33)	0.99 (0.83–1.18)	1.07 (0.85–1.36)
Model 2			
Metabolically-healthy, obesity	Reference	Reference	Reference
Metabolically-healthy, normal-weight	0.81 (0.71–0.92)	0.63 (0.51–0.77)	0.69 (0.53–0.91)
Metabolically-unhealthy, normal-weight	0.86 (0.76–0.98)	0.65 (0.53–0.79)	0.70 (0.54–0.90)
Metabolically-unhealthy, overweight	0.96 (0.84–1.09)	0.74 (0.61–0.89)	0.74 (0.58–0.95)
Metabolically-unhealthy, obesity	1.16 (1.02–1.31)	0.97 (0.81–1.15)	1.05 (0.83–1.33)
Model 3			
Metabolically-healthy, obesity	Reference	Reference	Reference
Metabolically-healthy, normal-weight	0.84 (0.74–0.97)	0.65 (0.53–0.81)	0.72 (0.55–0.94)
Metabolically-unhealthy, normal-weight	0.79 (0.69–0.90)	0.58 (0.48–0.71)	0.61 (0.47–0.80)
Metabolically-unhealthy, overweight	0.85 (0.74–0.97)	0.63 (0.52–0.77)	0.63 (0.48–0.81)
Metabolically-unhealthy, obesity	0.99 (0.86–1.13)	0.80 (0.66–0.97)	0.86 (0.67–1.10)

Values in parenthesis are 95% confidence intervals.

^a^ Compared to individuals without adenoma. Model 1: Adjusted for age and sex. Model 2: Further adjusted for smoking, alcohol, first-degree family history of colorectal cancer, and aspirin use. Model 3: Further adjusted for fasting blood glucose, systolic blood pressure, triglyceride, high-density lipoprotein cholesterol, low-density lipoprotein cholesterol, and HOMA-IR.

## Discussion

In this large study of asymptomatic Korean adults undergoing first-time screening colonoscopies, we found a significant positive association between BMI and CRA in metabolically-healthy population. The magnitude of association was even stronger for multiple CRAs and high-risk CRA. The association was independent of traditional risk factors for colorectal neoplasia, including metabolic mediators below levels considered abnormal. These findings indicate that excess adiposity increases CRC risk in metabolically-healthy population.

A large body of epidemiological data provides solid evidence on the relationship between obesity and diabetes [16], cardiovascular diseases [16, 17], and malignancies in different sites [18]. The key mechanisms linking obesity and most of its related complications are insulin resistance and metabolic syndrome. However, there are subjects with a metabolically benign fat distribution, without an increased insulin resistance, and who are considered to be healthy despite a high degree of obesity. Debate continues concerning whether individuals with MHO are truly healthy. The MHO population had a similar risk for cardiovascular events as the metabolically healthy, normal-weight population in some studies [19–21], but had an increased risk in others [22, 23]. Furthermore, a substantial proportion of the MHO population has developed deleterious metabolic changes associated with obesity over time [24, 25]. It has been reported that the MHO population has an increased risk of developing diabetes, chronic kidney disease, and nonalcoholic fatty liver disease [26–28].

In the present study, we demonstrated that the MHO phenotype was closely associated with an increased risk of CRA, including high-risk adenoma. Our findings are consistent with the previous study [13]. An analysis of 18,085 young Korean adults revealed that the prevalence of low-risk and high-risk CRA was increased in MHO individuals (OR = 1.44; 95% CI, 1.23–1.69 and OR = 1.62; 95% CI, 1.09–2.41, respectively) compared to normal, healthy individuals, after adjusting for age, sex, smoking, drinking, exercise, family history of CRC, education, and the use of analgesics and aspirin. The prevalence of low-risk and high-risk CRA was associated with increased categories of BMI in a dose-response manner. However, this study had a limitation that the enrolled participants were very young (mean ± SD age, 39.7 ± 6.8 years), which is far below 50 years, the recommended age for beginning to screen for CRC with substantial net benefit by guideline [29]. Screening at lower ages unavoidably led to a low detection rate of CRA (9.3% and 1.4% for low- and high-risk adenoma, respectively). In the present study, we only enrolled participants aged 40 years or older, and the overall prevalence of CRA was 25.6% in metabolically-healthy participants. Furthermore, we additionally adjusted for metabolic mediators, including fasting blood glucose, systolic blood pressure, triglyceride, HDL-C, LDL-C, and HOMA-IR, to account for possible mediation by these metabolic components below levels considered abnormal. Although we used strict criteria to define the metabolically healthy phenotype, and focused on subjects with no metabolic abnormalities and no insulin resistance, we found that even in this population, the values of metabolic parameters increased with increasing BMI. However, adjustment for metabolic parameters below levels considered abnormal did not significantly attenuate the association, suggesting that obesity, per se, irrespective of insulin resistance or metabolic abnormalities, is a crucial element linked to CRA. Recent evidence indicated that height is associated with higher cancer risk [30]. Insulin and insulin-like growth factor signaling pathways were suggested as one of mechanisms linking height to cancer. In our dataset, additional adjustment for height did not virtually affect the result (data not shown).

The mechanisms whereby obesity contributes to CRA remain incompletely elucidated. One major purported mechanism is the imbalance of adipokine profile [31]. Adipose tissue is a highly active player in the innate immune response, in which adipokine is responsible for a paracrine loop between adipocytes and macrophages. This interplay causes systemic chronic low-grade inflammation, providing a favorable niche for tumor development. Several studies have suggested that an inverse correlation exists between serum adiponectin and colorectal neoplasia [32–34]. Additionally, Fujisawa, et al. reported that adiponectin knockout mice are more susceptible to colorectal carcinogen [35]. Other pre-clinical studies have also demonstrated that leptin and adiponectin, secreting from adipocytes, are important players in CRC tumorigenesis [36, 37]. Therefore, in addition to systemic metabolic markers such as glucose and lipid profiles, inflammatory markers or adipokines which could represent local metabolism in adipose tissue might be considered to better define metabolic status.

Notably, when we analyzed the risk of CRA in metabolically-healthy population by sex, the prevalence of CRA was higher in men than women in all BMI groups (S2 Table). The odds ratios of CRA in subjects with MHO compared to subjects with MHNW were similar in the direction in men and in women; however, they were much higher in women than men. Several genetic and epigenetic factors have been suggested to explain sex-specific differences in CRC risk [38]. Insulin and insulin-like growth factor axis may act differently by sex in CRC carcinogenesis [39]. Our findings suggest that adiposity may impose a different risk on CRC by sex, which warrants further investigation.

We also observed that the risk of adenoma was higher in MUO compared to MHO participants (Table 5, model 2). In consistent with our findings, higher CRC risk for MUO compared to MHO has been reported [40]. CRC risk was different by metabolic parameters within same anthropometric category, suggesting that the combination of anthropometric measures with metabolic parameters may be useful for defining risk for CRC.

This study has some limitations that need to be considered in the interpretation. First, we used BMI as a measure of adiposity. However, BMI does not distinguish fat mass from lean mass, which may have led to an underestimation of the association between increased adiposity and CRA. Second, obesity is an independent predictor of inadequate bowel preparation for colonoscopies [41], so the prevalence of CRA in obese participants may be underestimated. Third, among 9,182 participants, only 11 cases of invasive cancer were identified, which was too small to perform the association study. Detailed information on smoking or alcohol intake was unavailable for substantial proportion of participants, which limited the analysis of dose-dependent effect of smoking or alcohol intake on CRA. Finally, our study participants were all Koreans in health check-up settings. They are likely to be highly-motivated for their health for any number of reasons. In addition, South Koreans show disparities in prevalence, location, and shape characteristic of colorectal neoplasia compared to Westerners [42]. Thus, generalizability to other populations needs to be demonstrated. Also, the cross-sectional design of the study cannot address causal relationships. However, our study has several strengths, including the large sample size, the use of carefully standardized clinical, endoscopic, and laboratory procedures, and the availability of carefully phenotyped participants with no metabolic abnormalities, all of which added to the strength of our findings.

In conclusion, we demonstrated that the MHO phenotype was associated with an increased prevalence of CRA. This finding supports the conclusion that MHO increases the risk of CRC. Clinicians should be aware of this risk in MHO individuals and counsel them accordingly.

## Supporting information

S1 Questionnaire In Korean. (PDF)

S2 Questionnaire In English. (PDF)

S1 Table Association between body mass index and colorectal adenoma. (DOCX)

S2 Table Prevalence and odd ratios of colorectal adenoma by body mass index category among metabolically-healthy men and women. (DOCX)

## Abbreviations

BMI: body mass index
CRA: colorectal adenoma
CRC: colorectal cancer
HDL-C: high-density lipoprotein cholesterol
HOMA-IR: homeostasis model assessment of insulin resistance
LDL-C: low-density lipoprotein cholesterol
MHNW: metabolically-healthy normal-weight
MHO: metabolically-healthy obese
MUNW: metabolically-unhealthy normal-weight
MUO: metabolically-unhealthy obese
MUOW: metabolically-unhealthy overweight
