# Supplementary material for: Metabolically-healthy obesity is associated with higher prevalence of colorectal adenoma
Source: PLoS One. 2017 Jun 21;12(6):e0179480. doi: 10.1371/journal.pone.0179480 (PMC5479542; doi:10.1371/journal.pone.0179480)
Supplement: S1 Questionnaire — (PDF) [file pone.0179480.s001.pdf]

스테
플러

0 0 0

SAMSUNG 삼성서울병원

의 학 정 밀 문 진

본 의학 문진은 귀하의 건강상태를 정확히 파악하고 건강 위험 요인을 규명하는데 매우 중요한 자료입니다. 각 항목들을 자세히 검토하신 후 정확하게 작성해 주시면 감사하겠습니다.
(한국의 무증상 성인을 대상으로 하는 귀중한 의학적 통계자료에 참고가 될 수 있습니다.
이를 원하지 않는 분께서는 접수 시 의사표시를 하여 주시기 바랍니다.)

※ 작성 방법 : 빈칸에 숫자로 기입하시고, 해당되는 ○ 에 ● 또는 ✔ 표시를 해주십시오.
귀하의 성명과 진료카드번호를 적어 주십시오.

(0-1) 성 명	

건 진 날 짜							
년 도 (0-2)				월 (0-3)		일 (0-4)	
2	0						
⓪	●	⓪	⓪	⓪	⓪	⓪	⓪
①	①	①	①	①	①	①	①
●	②	②	②		②	②	②
③	③	③	③		③	③	③
④	④	④	④		④		④
⑤	⑤	⑤	⑤		⑤		⑤
⑥	⑥	⑥	⑥		⑥		⑥
⑦	⑦	⑦	⑦		⑦		⑦
⑧	⑧	⑧	⑧		⑧		⑧
⑨	⑨	⑨	⑨		⑨		⑨

진 료 카 드 번 호 (0-5)							
⓪	⓪	⓪	⓪	⓪	⓪	⓪	⓪
①	①	①	①	①	①	①	①
②	②	②	②	②	②	②	②
③	③	③	③	③	③	③	③
	④	④	④	④	④	④	④
	⑤	⑤	⑤	⑤	⑤	⑤	⑤
	⑥	⑥	⑥	⑥	⑥	⑥	⑥
	⑦	⑦	⑦	⑦	⑦	⑦	⑦
	⑧	⑧	⑧	⑧	⑧	⑧	⑧
	⑨	⑨	⑨	⑨	⑨	⑨	⑨

건 강 의 학 센 터

스테
플러

001

※ 각 항목들을 자세히 검토하신 후 정확하게 작성해 주십시오.

(1-1)
1. 건강진단을 받으시려는 동기는 무엇입니까?

① 정기 검진 ② 최근 건강에 이상을 느껴서

(1-2)
☞ ②번으로 답하신 분은 증상을 적어 주십시오.

____

____

____

(1-3)
2. 귀하는 담배를 피우고 있거나 피우신 적이 있습니까?

① 아니오, 원래 안피운다.

② 과거에는 피웠지만 지난 1년 이상 담배를 전혀 피우지 않았다.

③ 예 (현재 피우고 있거나 금연한지 1년이 안되었다.)

▶ ②번과 ③번 이라고 답하신 분은 아래 문항에 표시하여 주십시오.

2-1. 처음 담배를 피우기 시작한 연령은? (1-4)

만____세	⓪ ① ② ③ ④ ⑤ ⑥ ⑦
	⓪ ① ② ③ ④ ⑤ ⑥ ⑦ ⑧ ⑨

2-2. 총 흡연 기간은? (1-5)

총____년	⓪ ① ② ③ ④ ⑤ ⑥ ⑦
	⓪ ① ② ③ ④ ⑤ ⑥ ⑦ ⑧ ⑨

(1-6)
2-3. 흡연을 하실 때 하루 평균 흡연량은?

① 10개비 이하 ② 11 ～ 20개비 ③ 21 ～ 30개비 ④ 31개비 이상

(1-7)
2-4. 몇 년 동안 금연하셨나요(해당하실 경우에만 기입)?

① 2년 이하 ② 3 ～ 4년 ③ 5 ～ 9년 ④ 10 ～ 14년 ⑤ 15년 이상

스테
플러

0 0 2

(2-1)
3. 귀하는 술을 드십니까?

① 아니오. ② 예

☞ **"② 예"라고 답하신 분은 아래 문항에 표기하여 주십시오.**

3-1. 지금까지 드신 총 기간은?

(2-2)

총_____년	⓪ ① ② ③ ④ ⑤ ⑥ ⑦ ⑧
	⓪ ① ② ③ ④ ⑤ ⑥ ⑦ ⑧ ⑨

(2-3)
3-2. 평균적으로 드시는 횟수는?

① 월1회 이하 ② 월2 ~ 3회 ③ 주1 ~ 2회 ④ 주3 ~ 4회 ⑤ 주5 ~ 6회 ⑥ 매일

(2-4)
3-3. 한 번 드실 때 주량은?(소주를 기준으로)

① 1 ~ 2잔 ② 반병 ③ 1병 ④ 2병 이상

(2-5)
4. 약물 부작용이 있습니까?

① 예 ② 아니오 ③ 모르겠다.

(2-6)
4-1. 약물 부작용이 있으시다면 그 원인은 무엇입니까?

① 아스피린 및 소염 진통제 ② 항생제 ③ 조영제 ④ 국소마취제 ⑤ 기타

(2-7)
5. 직장에서의 활동이나 레크리에이션, 운동, 스포츠 등 모든 활동을 포함하여 지난 7일 동안 주로 어떠한 신체적 활동을 하셨습니까?

한번에 10분 이상 지속한 신체 활동에 대해서 가장 근접한 한가지를 선택해 주십시오.

① 격렬한 신체활동 - 예)직업상 노동, 에어로빅, 빠른 자전거 타기, 조깅, 축구시합 등

② 중정도의 신체활동 - 예)보통속도의 자전거 타기, 속보, 복식테니스, 수영, 등산 등

③ 낮은 신체활동 - 예)걷기, 골프, 집안일 등

④ 신체활동량이 없다. - 한 번에 10분 이상 걸은 적이 없다.

(2-8)
5-1. 위의 신체활동을 일주일에 몇 회 정도 하고 계십니까?

① 없다 ② 1 ~ 2일 ③ 3 ~ 4일 ④ 5일 이상

(2-9)
5-2. 보통 하루의 신체활동량은 어떻게 되십니까?

① 없다 ② 20분 이하 ③ 20 ~ 40분 ④ 40 ~ 60분 ⑤ 60분 이상

0 0 3

스테플러

(3-1)

6. 귀하는 지금까지 의사로부터 다음의 질환을 진단 받으신 적이 있습니까(암은 제외)?

○ 진단받은 적이 없다.

진단 받으신 적이 있으시다면 아래 해당 항목에 표시하여 주십시오.

질 환 명	진단 및 치료 여부	수술 혹은 시술 여부	진 단 시 기 (연령)	
고혈압	○ 치료받은 적 없음<br>○ 과거에 치료했었음<br>○ 현재 치료중 (3-2)		만____세	(3-3)<br>① ② ③ ④ ⑤ ⑥ ⑦ ⑧ ⑨<br>⓪ ① ② ③ ④ ⑤ ⑥ ⑦ ⑧ ⑨
당뇨병	○ 치료받은 적 없음<br>○ 과거에 치료했었음<br>○ 현재 치료중 (3-4)		만____세	(3-5)<br>① ② ③ ④ ⑤ ⑥ ⑦ ⑧ ⑨<br>⓪ ① ② ③ ④ ⑤ ⑥ ⑦ ⑧ ⑨
고지혈증	○ 치료받은 적 없음<br>○ 과거에 치료했었음<br>○ 현재 치료중 (3-6)		만____세	(3-7)<br>① ② ③ ④ ⑤ ⑥ ⑦ ⑧ ⑨<br>⓪ ① ② ③ ④ ⑤ ⑥ ⑦ ⑧ ⑨
협심증	○ 치료받은 적 없음<br>○ 과거에 치료했었음<br>○ 현재 치료중 (3-8)	(3-9)<br>○ 예<br>○ 아니오	만____세	(3-10)<br>① ② ③ ④ ⑤ ⑥ ⑦ ⑧ ⑨<br>⓪ ① ② ③ ④ ⑤ ⑥ ⑦ ⑧ ⑨
심근경색	○ 치료받은 적 없음<br>○ 과거에 치료했었음<br>○ 현재 치료중 (3-11)	(3-12)<br>○ 예<br>○ 아니오	만____세	(3-13)<br>① ② ③ ④ ⑤ ⑥ ⑦ ⑧ ⑨<br>⓪ ① ② ③ ④ ⑤ ⑥ ⑦ ⑧ ⑨
뇌졸중/중풍	○ 치료받은 적 없음<br>○ 과거에 치료했었음<br>○ 현재 치료중 (3-14)	(3-15)<br>○ 예<br>○ 아니오	만____세	(3-16)<br>① ② ③ ④ ⑤ ⑥ ⑦ ⑧ ⑨<br>⓪ ① ② ③ ④ ⑤ ⑥ ⑦ ⑧ ⑨
B형 간염	○ 치료받은 적 없음<br>○ 과거에 치료했었음<br>○ 현재 치료중 (3-17)		만____세	(3-18)<br>① ② ③ ④ ⑤ ⑥ ⑦ ⑧ ⑨<br>⓪ ① ② ③ ④ ⑤ ⑥ ⑦ ⑧ ⑨
C형 간염	○ 치료받은 적 없음<br>○ 과거에 치료했었음<br>○ 현재 치료중 (3-19)		만____세	(3-20)<br>① ② ③ ④ ⑤ ⑥ ⑦ ⑧ ⑨<br>⓪ ① ② ③ ④ ⑤ ⑥ ⑦ ⑧ ⑨
간경변	○ 치료받은 적 없음<br>○ 과거에 치료했었음<br>○ 현재 치료중 (3-21)		만____세	(3-22)<br>① ② ③ ④ ⑤ ⑥ ⑦ ⑧ ⑨<br>⓪ ① ② ③ ④ ⑤ ⑥ ⑦ ⑧ ⑨
지방간	○ 치료받은 적 없음<br>○ 과거에 치료했었음<br>○ 현재 치료중 (3-23)		만____세	(3-24)<br>① ② ③ ④ ⑤ ⑥ ⑦ ⑧ ⑨<br>⓪ ① ② ③ ④ ⑤ ⑥ ⑦ ⑧ ⑨
위, 십이지장 궤양	○ 치료받은 적 없음<br>○ 과거에 치료했었음<br>○ 현재 치료중 (3-25)	(3-26)<br>○ 예<br>○ 아니오	만____세	(3-27)<br>① ② ③ ④ ⑤ ⑥ ⑦ ⑧ ⑨<br>⓪ ① ② ③ ④ ⑤ ⑥ ⑦ ⑧ ⑨
헬리코박터 균	○ 치료받은 적 없음<br>○ 과거에 치료했었음<br>○ 현재 치료중 (3-28)		만____세	(3-29)<br>① ② ③ ④ ⑤ ⑥ ⑦ ⑧ ⑨<br>⓪ ① ② ③ ④ ⑤ ⑥ ⑦ ⑧ ⑨

004

스테플러

질 환 명	진단 및 치료 여부	수술 혹은 시술 여부	진 단 시 기 (연령)	
대장폴립 (용종)	◯ 치료받은 적 없음<br>◯ 과거에 치료했었음<br>◯ 현재 치료중 (4-1)	(4-2)<br>◯ 예<br>◯ 아니오	만____세	(4-3)<br>① ② ③ ④ ⑤ ⑥ ⑦ ⑧ ⑨<br>⓪ ① ② ③ ④ ⑤ ⑥ ⑦ ⑧ ⑨
만성 폐쇄성 폐질환	◯ 치료받은 적 없음<br>◯ 과거에 치료했었음<br>◯ 현재 치료중 (4-4)		만____세	(4-5)<br>① ② ③ ④ ⑤ ⑥ ⑦ ⑧ ⑨<br>⓪ ① ② ③ ④ ⑤ ⑥ ⑦ ⑧ ⑨
천식	◯ 치료받은 적 없음<br>◯ 과거에 치료했었음<br>◯ 현재 치료중 (4-6)		만____세	(4-7)<br>① ② ③ ④ ⑤ ⑥ ⑦ ⑧ ⑨<br>⓪ ① ② ③ ④ ⑤ ⑥ ⑦ ⑧ ⑨
결핵	◯ 치료받은 적 없음<br>◯ 과거에 치료했었음<br>◯ 현재 치료중 (4-8)	(4-9)<br>◯ 예<br>◯ 아니오	만____세	(4-10)<br>① ② ③ ④ ⑤ ⑥ ⑦ ⑧ ⑨<br>⓪ ① ② ③ ④ ⑤ ⑥ ⑦ ⑧ ⑨
유방양성종양	◯ 치료받은 적 없음<br>◯ 과거에 치료했었음<br>◯ 현재 치료중 (4-11)	(4-12)<br>◯ 예<br>◯ 아니오	만____세	(4-13)<br>① ② ③ ④ ⑤ ⑥ ⑦ ⑧ ⑨<br>⓪ ① ② ③ ④ ⑤ ⑥ ⑦ ⑧ ⑨
갑상선 기능 저하증 및 항진증	◯ 치료받은 적 없음<br>◯ 과거에 치료했었음<br>◯ 현재 치료중 (4-14)	(4-15)<br>◯ 예<br>◯ 아니오	만____세	(4-16)<br>① ② ③ ④ ⑤ ⑥ ⑦ ⑧ ⑨<br>⓪ ① ② ③ ④ ⑤ ⑥ ⑦ ⑧ ⑨
갑상선 결절 및 낭종	◯ 치료받은 적 없음<br>◯ 과거에 치료했었음<br>◯ 현재 치료중 (4-17)	(4-18)<br>◯ 예<br>◯ 아니오	만____세	(4-19)<br>① ② ③ ④ ⑤ ⑥ ⑦ ⑧ ⑨<br>⓪ ① ② ③ ④ ⑤ ⑥ ⑦ ⑧ ⑨
신장 및 방광 질환	◯ 치료받은 적 없음<br>◯ 과거에 치료했었음<br>◯ 현재 치료중 (4-20)	(4-21)<br>◯ 예<br>◯ 아니오	만____세	(4-22)<br>① ② ③ ④ ⑤ ⑥ ⑦ ⑧ ⑨<br>⓪ ① ② ③ ④ ⑤ ⑥ ⑦ ⑧ ⑨
신장 요로 결석	◯ 치료받은 적 없음<br>◯ 과거에 치료했었음<br>◯ 현재 치료중 (4-23)	(4-24)<br>◯ 예<br>◯ 아니오	만____세	(4-25)<br>① ② ③ ④ ⑤ ⑥ ⑦ ⑧ ⑨<br>⓪ ① ② ③ ④ ⑤ ⑥ ⑦ ⑧ ⑨
혈뇨	◯ 치료받은 적 없음<br>◯ 과거에 치료했었음<br>◯ 현재 치료중 (4-26)	(4-27)<br>◯ 예<br>◯ 아니오	만____세	(4-28)<br>① ② ③ ④ ⑤ ⑥ ⑦ ⑧ ⑨<br>⓪ ① ② ③ ④ ⑤ ⑥ ⑦ ⑧ ⑨
백내장	◯ 치료받은 적 없음<br>◯ 과거에 치료했었음<br>◯ 현재 치료중 (4-29)	(4-30)<br>◯ 예<br>◯ 아니오	만____세	(4-31)<br>① ② ③ ④ ⑤ ⑥ ⑦ ⑧ ⑨<br>⓪ ① ② ③ ④ ⑤ ⑥ ⑦ ⑧ ⑨
녹내장	◯ 치료받은 적 없음<br>◯ 과거에 치료했었음<br>◯ 현재 치료중 (4-32)	(4-33)<br>◯ 예<br>◯ 아니오	만____세	(4-34)<br>① ② ③ ④ ⑤ ⑥ ⑦ ⑧ ⑨<br>⓪ ① ② ③ ④ ⑤ ⑥ ⑦ ⑧ ⑨
디스크 (허리/목)	◯ 치료받은 적 없음<br>◯ 과거에 치료했었음<br>◯ 현재 치료중 (4-35)	(4-36)<br>◯ 예<br>◯ 아니오	만____세	(4-37)<br>① ② ③ ④ ⑤ ⑥ ⑦ ⑧ ⑨<br>⓪ ① ② ③ ④ ⑤ ⑥ ⑦ ⑧ ⑨
(4-38)<br>기타<br>(__________)	◯ 치료받은 적 없음<br>◯ 과거에 치료했었음<br>◯ 현재 치료중 (4-39)	(4-40)<br>◯ 예<br>◯ 아니오	만____세	(4-41)<br>① ② ③ ④ ⑤ ⑥ ⑦ ⑧ ⑨<br>⓪ ① ② ③ ④ ⑤ ⑥ ⑦ ⑧ ⑨

스테
플러

0 0 5

(5-1)
7. 현재 한 달 이상 지속적으로 복용하고 계신 약이 있습니까(패치와 흡입제 포함)?

◯ 지속적으로 복용하는 약이 없다.

지속적으로 복용하는 약이 있으시다면 아래 해당 항목에 표시하여 주십시오.

약 이 름	치 료 여 부	약 이 름	치 료 여 부
혈압약	(5-2) ◯ 현재 치료중 ◯ 과거 치료	갑상선 치료제	(5-3) ◯ 현재 치료중 ◯ 과거 치료
당뇨약(경구약 및 인슐린)	(5-4) ◯ 현재 치료중 ◯ 과거 치료	골다공증약	(5-5) ◯ 현재 치료중 ◯ 과거 치료
고지혈증약	(5-6) ◯ 현재 치료중 ◯ 과거 치료	여성 호르몬제	(5-7) ◯ 현재 치료중 ◯ 과거 치료
아스피린	(5-8) ◯ 현재 치료중 ◯ 과거 치료	남성 호르몬제	(5-9) ◯ 현재 치료중 ◯ 과거 치료
와파린	(5-10) ◯ 현재 치료중 ◯ 과거 치료	칼슘 제재	(5-11) ◯ 현재 치료중 ◯ 과거 치료
기타 혈전 방지제	(5-12) ◯ 현재 치료중 ◯ 과거 치료	철분 제재	(5-13) ◯ 현재 치료중 ◯ 과거 치료
부정맥약	(5-14) ◯ 현재 치료중 ◯ 과거 치료	수면제, 항우울증약<br>기타 신경정신과 약물	(5-15) ◯ 현재 치료중 ◯ 과거 치료
위장약<br>(소화제, 제산제)	(5-16) ◯ 현재 치료중 ◯ 과거 치료	호흡기 약물<br>(흡입제 포함)	(5-17) ◯ 현재 치료중 ◯ 과거 치료
간장약<br>(간보호제)	(5-18) ◯ 현재 치료중 ◯ 과거 치료	영양제 및 보조 식품	(5-19) ◯ 현재 치료중 ◯ 과거 치료
변비약	(5-20) ◯ 현재 치료중 ◯ 과거 치료	한약, 보약	(5-21) ◯ 현재 치료중 ◯ 과거 치료
소염 진통제<br>(관절염약, 두통약)	(5-22) ◯ 현재 치료중 ◯ 과거 치료	기타 약제	(5-23) ◯ 현재 치료중 ◯ 과거 치료

스테
플러

0 0 6

(6-1)
8. 지금까지 암에 걸리신 적이 있습니까?

○ 암에 걸린 적이 없다.

암에 걸린 적이 있으시다면 아래 해당 항목에 표시하여 주십시오.

암 종 류	치료 방법<br>(중복 기입 가능)	진단 받은 연령	
(6-2)<br>○ 위암	(6-3)<br>○ 수술 ○ 약물치료<br>○ 방사선치료 ○ 기타 다른 치료	만_____세	(6-4)<br>① ② ③ ④ ⑤ ⑥ ⑦ ⑧ ⑨<br>⓪ ① ② ③ ④ ⑤ ⑥ ⑦ ⑧ ⑨
(6-5)<br>○ 폐암	(6-6)<br>○ 수술 ○ 약물치료<br>○ 방사선치료 ○ 기타 다른 치료	만_____세	(6-7)<br>① ② ③ ④ ⑤ ⑥ ⑦ ⑧ ⑨<br>⓪ ① ② ③ ④ ⑤ ⑥ ⑦ ⑧ ⑨
(6-8)<br>○ 간암	(6-9)<br>○ 수술 ○ 약물치료<br>○ 방사선치료 ○ 기타 다른 치료	만_____세	(6-10)<br>① ② ③ ④ ⑤ ⑥ ⑦ ⑧ ⑨<br>⓪ ① ② ③ ④ ⑤ ⑥ ⑦ ⑧ ⑨
(6-11)<br>○ 대장암	(6-12)<br>○ 수술 ○ 약물치료<br>○ 방사선치료 ○ 기타 다른 치료	만_____세	(6-13)<br>① ② ③ ④ ⑤ ⑥ ⑦ ⑧ ⑨<br>⓪ ① ② ③ ④ ⑤ ⑥ ⑦ ⑧ ⑨
(6-14)<br>○ 유방암	(6-15)<br>○ 수술 ○ 약물치료<br>○ 방사선치료 ○ 기타 다른 치료	만_____세	(6-16)<br>① ② ③ ④ ⑤ ⑥ ⑦ ⑧ ⑨<br>⓪ ① ② ③ ④ ⑤ ⑥ ⑦ ⑧ ⑨
(6-17)<br>○ 자궁경부암	(6-18)<br>○ 수술 ○ 약물치료<br>○ 방사선치료 ○ 기타 다른 치료	만_____세	(6-19)<br>① ② ③ ④ ⑤ ⑥ ⑦ ⑧ ⑨<br>⓪ ① ② ③ ④ ⑤ ⑥ ⑦ ⑧ ⑨
(6-20)<br>○ 갑상선암	(6-21)<br>○ 수술 ○ 약물치료<br>○ 방사선치료 ○ 기타 다른 치료	만_____세	(6-22)<br>① ② ③ ④ ⑤ ⑥ ⑦ ⑧ ⑨<br>⓪ ① ② ③ ④ ⑤ ⑥ ⑦ ⑧ ⑨
(6-23)<br>○ 방광암	(6-24)<br>○ 수술 ○ 약물치료<br>○ 방사선치료 ○ 기타 다른 치료	만_____세	(6-25)<br>① ② ③ ④ ⑤ ⑥ ⑦ ⑧ ⑨<br>⓪ ① ② ③ ④ ⑤ ⑥ ⑦ ⑧ ⑨
(6-26)<br>○ 식도암	(6-27)<br>○ 수술 ○ 약물치료<br>○ 방사선치료 ○ 기타 다른 치료	만_____세	(6-28)<br>① ② ③ ④ ⑤ ⑥ ⑦ ⑧ ⑨<br>⓪ ① ② ③ ④ ⑤ ⑥ ⑦ ⑧ ⑨
(6-29)<br>○ 담낭(쓸개)암<br>또는 담도암	(6-30)<br>○ 수술 ○ 약물치료<br>○ 방사선치료 ○ 기타 다른 치료	만_____세	(6-31)<br>① ② ③ ④ ⑤ ⑥ ⑦ ⑧ ⑨<br>⓪ ① ② ③ ④ ⑤ ⑥ ⑦ ⑧ ⑨
(6-32)<br>○ 난소암	(6-33)<br>○ 수술 ○ 약물치료<br>○ 방사선치료 ○ 기타 다른 치료	만_____세	(6-34)<br>① ② ③ ④ ⑤ ⑥ ⑦ ⑧ ⑨<br>⓪ ① ② ③ ④ ⑤ ⑥ ⑦ ⑧ ⑨
(6-35)<br>○ 전립선암	(6-36)<br>○ 수술 ○ 약물치료<br>○ 방사선치료 ○ 기타 다른 치료	만_____세	(6-37)<br>① ② ③ ④ ⑤ ⑥ ⑦ ⑧ ⑨<br>⓪ ① ② ③ ④ ⑤ ⑥ ⑦ ⑧ ⑨
(6-38)<br>○ 췌장암	(6-39)<br>○ 수술 ○ 약물치료<br>○ 방사선치료 ○ 기타 다른 치료	만_____세	(6-40)<br>① ② ③ ④ ⑤ ⑥ ⑦ ⑧ ⑨<br>⓪ ① ② ③ ④ ⑤ ⑥ ⑦ ⑧ ⑨
(6-41)<br>○ 기타암<br>(6-42)<br>(____________)	(6-43)<br>○ 수술 ○ 약물치료<br>○ 방사선치료 ○ 기타 다른 치료	만_____세	(6-44)<br>① ② ③ ④ ⑤ ⑥ ⑦ ⑧ ⑨<br>⓪ ① ② ③ ④ ⑤ ⑥ ⑦ ⑧ ⑨

스테
플러

007

(7-1)
9. 가족이나 친척이 다음의 질환이나 암을 진단받은 적이 있습니까?

○ 진단 받은 적이 없다.

진단 받으신 분이 있으시다면 아래 해당 항목에 표시하여 주십시오.

질 환	부	모	형제	자녀	조부	조모	외조부	외조모
고혈압 (7-2)	○	○	○	○	○	○	○	○
당뇨병 (7-3)	○	○	○	○	○	○	○	○
심근경색/협심증 (7-4)	○	○	○	○	○	○	○	○
뇌졸중(뇌경색,뇌출혈) (7-5)	○	○	○	○	○	○	○	○
치매 (7-6)	○	○	○	○	○	○	○	○
만성간염 또는 간경변 (7-7)	○	○	○	○	○	○	○	○
천식 또는 만성 폐쇄성 질환 (7-8)	○	○	○	○	○	○	○	○
위암 (7-9)	○	○	○	○	○	○	○	○
폐암 (7-10)	○	○	○	○	○	○	○	○
간암 (7-11)	○	○	○	○	○	○	○	○
대장암 (7-12)	○	○	○	○	○	○	○	○
유방암 (7-13)	○	○	○	○	○	○	○	○
자궁경부암 (7-14)	○	○	○	○	○	○	○	○
갑상선암 (7-15)	○	○	○	○	○	○	○	○
방광암 (7-16)	○	○	○	○	○	○	○	○
식도암 (7-17)	○	○	○	○	○	○	○	○
담낭(쓸개)암 또는 담도암 (7-18)	○	○	○	○	○	○	○	○
난소암 (7-19)	○	○	○	○	○	○	○	○
전립선암 (7-20)	○	○	○	○	○	○	○	○
췌장암 (7-21)	○	○	○	○	○	○	○	○
기타암 (7-22)	○	○	○	○	○	○	○	○

스테
플러

008

계 통 별 설 문

최근에 아래와 같은 증상이 있으시면 표시해 주십시오.

(8-1)
1. 소화기

- ○ 음식을 삼키기가 힘들고 아프다.
- ○ 신물이 넘어온다.
- ○ 구역질이나 구토가 난다.
- ○ 소화가 잘 안된다.
- ○ 속이 쓰리다.
- ○ 대변이 자장면처럼 검게 나온다.
- ○ 대변에 붉은 피가 섞여 나온다.
- ○ 설사가 자주 난다.
- ○ 변비가 있다.
- ○ 변이 연필처럼 가늘게 나온다.
- ○ 배에 덩어리가 만져진다.
- ○ 배가 자주 아프다.

(8-2)
2. 호흡기

- ○ 최근 기침이 잦다.
- ○ 황색 혹은 녹색 가래가 나온다.
- ○ 객혈한 적이 있다.
- ○ 숨쉴 때 가슴에서 쌕쌕 소리가 난다.
- ○ 조금만 활동하여도 숨이 차다.

(8-3)
3. 신장·요로·비뇨기

- ○ 최근 소변량이 많아졌다.
- ○ 소변 보기가 힘들고 잔뇨감이 있다.
- ○ 소변을 참지 못하겠다.
- ○ 소변을 볼 때 아프다.
- ○ 소변 줄기가 가늘어졌다.
- ○ 소변이 빨갛거나 콜라색으로 나온다.
- ○ 옆구리나 아랫배에 심한 통증이 있다.
- ○ 잠을 자는 동안 소변을 보려고 자주 깬다.
- ○ 자신도 모르게 소변이 흘러 나온다
- ○ 성생활에 문제가 있다.

(8-4)
4. 심장·혈관

- ○ 가슴이 조여들며 통증이 팔이나 등으로 뻗치는 일이 있다.
- ○ 운동할 때 전과 달리 가슴이 답답해지거나 숨이 몹시 차다.
- ○ 갑자기 가슴이 두근거리거나 맥박이 불규칙해질 때가 있다.
- ○ 얼굴이나 손발이 자주 붓는다.
- ○ 잘 때나 누울 때 가슴이 답답하고 숨이 차며 앉으면 오히려 편해진다.
- ○ 걸으면 종아리가 아파서 쉬어야 한다.

(8-5)
5. 대사·내분비

- ○ 최근 식욕이 없다.
- ○ 손·발톱이나 머리카락이 잘 부러진다.
- ○ 자주 갈증이 난다.
- ○ 자주 얼굴이 화끈 달아오른다.
- ○ 추위를 많이 탄다.
- ○ 더위를 많이 탄다.

(8-6)
6. 신경·정신

- ○ 현기증이 자주 난다.
- ○ 두통이 있다.
- ○ 졸도한 경험이 있다.
- ○ 신체에 마비가 온 적이 있다.
- ○ 손발이 계속 저리다.
- ○ 행동이 느려지거나, 손이 떨린다.
- ○ 집중력, 기억력이 떨어진다.
- ○ 불안, 초조, 울적하다.
- ○ 가끔 주위가 빙빙 돈다.

0 0 9

스테플러

(9-1)
7. 근 골격

- ◯ 허리가 아프다.
- ◯ 무릎이 아프다.
- ◯ 어깨가 아프다.
- ◯ 뒷목이 뻐근하다.
- ◯ 뼈마디가 쑤시고 아프다.
- ◯ 관절이 아프거나 부은 적이 있다.
- ◯ 관절 운동에 장애가 있다.

(9-2)
8. 치아

- ◯ 치아나 구강 내에 통증이 있다.
- ◯ 양치질시 피가 난다.
- ◯ 구취가 난다.
- ◯ 스케일링을 1년에 1번이상 받는다.
- ◯ 임플란트 치료를 받았다.
- ◯ 턱관절 통증이 있다.

(9-3)
9. 기타

- ◯ 특별한 이유없이 체중이 준다 (최근 6개월간 평상시 체중의 10% 이상 감소).
- ◯ 쉽게 피로해진다.
- ◯ 기운이 없다.
- ◯ 열이나 오한이 난다.
- ◯ 수면 장애가 있다.
- ◯ 멍이 잘 들고 코피가 자주 난다.
- ◯ 피부가 몹시 가렵다.
- ◯ 피부에 발진이 있다.
- ◯ 두드러기가 잘 생긴다.
- ◯ 최근에 시력이 떨어졌다.
- ◯ 물체가 갑자기 두개로 보인다.
- ◯ 귀에서 소리가 난다.
- ◯ 2주 이상 목소리가 쉬었다.

스테플러

0 1 0

남 성 문 진

국제 전립선 증상 점수표

질 문 \ 점 수	전혀없다	5회 중 1회 이하	2회 중 1회 이하	절반정도	절반이상	거의항상
	0점	1점	2점	3점	4점	5점
(10-1) 1. 최근 한 달간 배뇨 후 시원치 않고 소변이 남아 있는 느낌이 얼마나 자주 있었습니까?	○	○	○	○	○	○
(10-2) 2. 최근 한 달간 배뇨 후 2시간 이내에 다시 소변을 보는 경우가 얼마나 자주 있었습니까?	○	○	○	○	○	○
(10-3) 3. 최근 한 달간 한번 소변을 볼때마다 소변줄기가 여러번 끊어진 경우가 얼마나 자주 있었습니까?	○	○	○	○	○	○
(10-4) 4. 최근 한 달간 소변이 마려울 때 참기 어려운 경우가 얼마나 자주 있었습니까?	○	○	○	○	○	○
(10-5) 5. 최근 한 달간 소변줄기가 약하다고 느낀 경우가 얼마나 자주 있었습니까?	○	○	○	○	○	○
(10-6) 6. 최근 한 달간 소변을 볼 때 금방 나오지 않아 힘을 주어야 하는 경우가 얼마나 있었습니까?	○	○	○	○	○	○
(10-7) 7. 최근 한 달간 밤에 잠을 자다가 소변을 보기 위해 몇 번이나 일어나십니까?	전혀없다	1회	2회	3회	4회	5회이상
	○	○	○	○	○	○

생 활 만 족 도

질 문 \ 점 수	0점	1점	2점	3점	4점	5점	6점
	○	○	○	○	○	○	○
(10-8) 8. 만약 지금같은 배뇨상태가 지속된다면 어떤 느낌이 드십니까?	매우 만족한다	만족한다	대체로 만족한다	만족, 불만족 반반이다	대체로 불만족한다	불만이다	매우 불만족한다

0 1 1

여 성 문 진

(11-1)

1. 초경은 언제 하셨습니까?

10세 이하	11세	12세	13세	14세	15세	16세	17세	18세	19세
○	○	○	○	○	○	○	○	○	○

(11-2)

2. 생리가 1년 이상 없습니까?

① 예 ② 아니오 ③ 있다가 없다가 한다. ④ 아니오, 전과 같이 나옵니다.

☞ "① 예"라고 답하신 분은 아래 문항에 표시해 주십시오.

2-1. 언제부터 나오지 않았습니까?

만______세	① ② ③ ④ ⑤ ⑥ ⑦<br>⓪ ① ② ③ ④ ⑤ ⑥ ⑦ ⑧ ⑨ (11-3)

(11-4)

2-2. 원인은 무엇입니까?

① 나이가 들어서 자연적으로 ② 자궁 적출술 후 ③ 양쪽 난소 절제술 후
④ 방사선 치료 후 ⑤ 약물요법 후

3. 여성호르몬 치료(복용약, 주사, 패치, 크림, 질정)를 받으신 적이 있으신 분은 아래 문항에 표시해 주십시오.

(11-5)

3-1. 총 치료 기간은?

① 1년 미만 ② 1년~3년 미만 ③ 3년~5년 미만
④ 5년~10년 미만 ⑤ 10년 이상

(11-6)

3-2. 현재도 치료 중이십니까?

① 예 ② 아니오, 끊었다.

스테플러

0 1 2

4. 임신한 적이 있으시다면 아래 문항에 표시해 주십시오.

4-1. 총 분만 횟수는?

(12-1)

횟 수	1회	2회	3회	4회	5회	6회	7회	8회	9회	10회 이상
총_______회	○	○	○	○	○	○	○	○	○	○

4-2. 어떤 방법으로 분만하셨습니까?

(12-2)

횟 수	1회	2회	3회	4회	5회	6회	7회	8회	9회	10회 이상
자연_______회	○	○	○	○	○	○	○	○	○	○

(12-3)

횟 수	1회	2회	3회	4회	5회 이상
제왕절개_______회	○	○	○	○	○

(12-4)

4-3. 유산 경험이 있으십니까?

① 있다 ② 없다

☞ 4-3-1. 유산경험이 있으시다면 횟수는?

(12-5)

횟 수	1회	2회	3회	4회	5회	6회	7회	8회	9회	10회 이상
자연_______회	○	○	○	○	○	○	○	○	○	○

(12-6)

횟 수	1회	2회	3회	4회	5회	6회	7회	8회	9회	10회 이상
인공_______회	○	○	○	○	○	○	○	○	○	○

(12-7)

4-4. 조산 경험이 있으십니까?

① 있다 ② 없다

☞ 4-4-1. 조산경험이 있으시다면 횟수는?

(12-8)

횟 수	1회	2회	3회	4회	5회	6회	7회	8회	9회	10회 이상
총_______회	○	○	○	○	○	○	○	○	○	○

스테
플러

0 1 3

스 트 레 스

※ 아래의 질문은 최근 몇 주 동안 경험하셨거나 느끼셨던 육체적 심리적 상태에 대한 것입니다. 해당되는 부분을 표시해 주십시오.

항 목	항상 그렇다	대부분 그렇다	약간 (가끔) 그렇다	전혀 그렇지 않다
(13-1) 1. 현재 매우 편안하며 건강하다고 느낀다.	○	○	○	○
(13-2) 2. 잠자고 난 후에도 개운한 감이 없다.	○	○	○	○
(13-3) 3. 매우 피곤하고 지쳐 있어 먹는 것 조차도 힘들다고 느낀다.	○	○	○	○
(13-4) 4. 근심걱정 때문에 편안하게 잠을 자지 못한다.	○	○	○	○
(13-5) 5. 정신이 맑고 깨끗하다고 느낀다.	○	○	○	○
(13-6) 6. 기력(원기)이 왕성함을 느낀다.	○	○	○	○
(13-7) 7. 밤이면 심란해 지거나 불안해 진다.	○	○	○	○
(13-8) 8. 대다수의 사람들과 마찬가지로 나를 잘 관리해 나간다고 생각한다.	○	○	○	○
(13-9) 9. 전체적으로 현재 내가 하고 있는 일은 잘 되어가고 있다고 느낀다.	○	○	○	○
(13-10) 10. 내가 행한 일의 방법이나 절차에 만족한다.	○	○	○	○
(13-11) 11. 어떤 일에 바로 착수(시작)할 수 있다.	○	○	○	○
(13-12) 12. 정상적인 일상생활을 즐길 수 있다.	○	○	○	○
(13-13) 13. 안절부절 못하거나 성질이 심술궂게 되어진다.	○	○	○	○
(13-14) 14. 나에게 닥친 문제를 해결해 나갈 수 있다.	○	○	○	○
(13-15) 15. 불행하고 우울함을 느낀다.	○	○	○	○
(13-16) 16. 나 자신에 대한 신뢰감이 없어지고 있다.	○	○	○	○
(13-17) 17. 모든 것을 고려해 볼 때 행복감을 느낀다.	○	○	○	○
(13-18) 18. 삶을 살아갈 만한 가치가 있다고 느낀다.	○	○	○	○

(13-19)
1. 요즈음 직장 때문에 스트레스를 어느 정도 받고 있다고 생각하십니까?
① 전혀 없다 ② 약간 ③ 보통 ④ 많이 ⑤ 매우 많다

(13-20)
2. 요즈음 집안 일(부부, 자녀, 가족관계)로 걱정되거나 우울한 일이 얼마나 있습니까?
① 전혀 없다 ② 약간 ③ 보통 ④ 많이 ⑤ 매우 많다

(13-21)
3. 스트레스 관리 상담을 원하십니까?
① 예 ② 아니오
