# Supplementary material for: Metabolically-healthy obesity is associated with higher prevalence of colorectal adenoma
Source: PLoS One. 2017 Jun 21;12(6):e0179480. doi: 10.1371/journal.pone.0179480 (PMC5479542; doi:10.1371/journal.pone.0179480)
Supplement: S2 Questionnaire — (PDF) [file pone.0179480.s002.pdf]

SAMSUNG **SAMSUNG MEDICAL CENTER**

Precision Medical Examination Questionnaire

This medical questionnaire is important material for checking up your health and analyzing the causes which threaten your health. Please check the following questions, and answer all of them.
(This survey will be used for the medical statistical data about foreigners who do not have any symptoms. If you do not want, please notify when you register.)

※ How to answer : Fill in the blank with the numbers,
and check with ● or ∨ on the ○ which is applicable to you.

**Name**	

**Date of Exam**							
**Year**				**Month**		**Day**	
2	0						
⓪	●	⓪	⓪	⓪	⓪		⓪
①	①	①	①	①	①		①
●	②	②	②		②		②
③	③	③	③		③		③
④	④	④	④		④		④
⑤	⑤	⑤	⑤		⑤		⑤
⑥	⑥	⑥	⑥		⑥		⑥
⑦	⑦	⑦	⑦		⑦		⑦
⑧	⑧	⑧	⑧		⑧		⑧
⑨	⑨	⑨	⑨		⑨		⑨

**Hospital ID No.**							
⓪	⓪	⓪	⓪	⓪	⓪	⓪	⓪
①	①	①	①	①	①	①	①
②	②	②	②	②	②	②	②
③	③	③	③	③	③	③	③
	④	④	④	④	④	④	④
	⑤	⑤	⑤	⑤	⑤	⑤	⑤
	⑥	⑥	⑥	⑥	⑥	⑥	⑥
	⑦	⑦	⑦	⑦	⑦	⑦	⑦
	⑧	⑧	⑧	⑧	⑧	⑧	⑧
	⑨	⑨	⑨	⑨	⑨	⑨	⑨

※Please read the following carefully and fill out the blank.

1. What is your purpose for participation in this screening program?

① Regular check up ② Recent health problems

☞ **if your answer is ②, describe the problems in detail.**

____

____

____

2. Have you ever smoked cigarettes?

① No, I've never smoked.

② I used to smoke, but I did not smoke in the past year.<br>③ Yes. (I smoke or it has not been a year since I quit smoking.)	**Please answer the followings, if your answer is ② or ③.**

2-1. How old were you when you started to smoke?	____year-old	ⓞ ① ② ③ ④ ⑤ ⑥ ⑦<br>ⓞ ① ② ③ ④ ⑤ ⑥ ⑦ ⑧ ⑨
2-2. How many years have you smoked?	_____year(s)	ⓞ ① ② ③ ④ ⑤ ⑥ ⑦<br>ⓞ ① ② ③ ④ ⑤ ⑥ ⑦ ⑧ ⑨

2-3. On average, how many cigarettes do you smoke per day?

① under 10 ② 11~20 ③ 21~30 ④ over 31

2-4. If you have quit, how many years are passed since you quit smoking? (If an attempt of smoking cessation was made for many times, mark the recent non-smoking period.)

① less than 2 yrs ② 3~4 yrs ③ 5~9 yrs ④ 10~14 yrs ⑤ over 15 yrs

3. Do you drink alcoholic beverages?

① No ② Yes

☞ **Please answer the followings if your answer is ②Yes**

3-1. How long have you drunk since you started to drink?

_____year(s)	ⓞ ① ② ③ ④ ⑤ ⑥ ⑦<br>ⓞ ① ② ③ ④ ⑤ ⑥ ⑦ ⑧ ⑨

3-2. On average, how often do you drink?

① once a month ② 2~3 times per month ③ 1~2 times per week
④ 3~4 times per week ⑤ 5~6 times per week ⑥ everyday

3-3. On average, how many bottles do you drink at once? (*bt=bottle)

① under ½ bt ② ½ bt ③ 1 bt ④ over 2 bt

4. Have you ever had a bad reaction to any medication?

① Yes ② No ③ Not sure

4-1. If yes, what is that medication?

① Aspirin or antibiotics ② Penicillin ③ Contrast agent
④ Local anesthetic ⑤ Others ( )

5. What kind of physical activities have you done for the last 7 days including exercise, sports activities, activities at the daily work place, and so on?

Please answer the most proper one about the physical activities that you've done for more than 10 minutes.

① Vigorous activities - ex) Hiking, Jogging, Basketball, Soccer, Bicycling fast, Carrying heavy loads, etc.
② Moderate activities - ex) Walking fast, Cleaning-heavy, Bicycling-light effort, Badminton, Tennis-double, etc.
③ Light activities - ex) Walking slowly, Standing-light works(cooking, washing dishes), etc.
④ None - I do not even walk for 10 minutes.

5-1. On average, how often do you do the above mentioned physical activities per week?

① None ② 1~2 days ③ 3~4 days ④ More than 5days

5-2. On average, how long do you do the physical activities per day?

① None ② Less than 20 min. ③ 20~40 min. ④ 40~60 min. ⑤ Over 60 min.

6. Have you ever been diagnosed with any of the followings?

○ No, I have never been diagnosed with any of the following disease.

If you have, please check the followings.

Diagnosis	Treatment	Surgery	The time of diagnosis (age)	
Hypertension (High blood pressure)	○ Never received treatment<br>○ Recieved treatment in the past<br>○ Currently on treatment		____yrs old	① ② ③ ④ ⑤ ⑥ ⑦ ⑧ ⑨<br>⓪ ① ② ③ ④ ⑤ ⑥ ⑦ ⑧ ⑨
Diabetes	○ Never received treatment<br>○ Recieved treatment in the past<br>○ Currently on treatment		____yrs old	① ② ③ ④ ⑤ ⑥ ⑦ ⑧ ⑨<br>⓪ ① ② ③ ④ ⑤ ⑥ ⑦ ⑧ ⑨
Dyslipidemia (High cholesterol, Triglyceride, etc.)	○ Never received treatment<br>○ Recieved treatment in the past<br>○ Currently on treatment		____yrs old	① ② ③ ④ ⑤ ⑥ ⑦ ⑧ ⑨<br>⓪ ① ② ③ ④ ⑤ ⑥ ⑦ ⑧ ⑨
Angina	○ Never received treatment<br>○ Recieved treatment in the past<br>○ Currently on treatment	○ Yes<br>○ No	____yrs old	① ② ③ ④ ⑤ ⑥ ⑦ ⑧ ⑨<br>⓪ ① ② ③ ④ ⑤ ⑥ ⑦ ⑧ ⑨
Myocardial infarction or Heart attack	○ Never received treatment<br>○ Recieved treatment in the past<br>○ Currently on treatment	○ Yes<br>○ No	____yrs old	① ② ③ ④ ⑤ ⑥ ⑦ ⑧ ⑨<br>⓪ ① ② ③ ④ ⑤ ⑥ ⑦ ⑧ ⑨
Stroke / Paralysis	○ Never received treatment<br>○ Recieved treatment in the past<br>○ Currently on treatment	○ Yes<br>○ No	____yrs old	① ② ③ ④ ⑤ ⑥ ⑦ ⑧ ⑨<br>⓪ ① ② ③ ④ ⑤ ⑥ ⑦ ⑧ ⑨
Hepatitis B	○ Never received treatment<br>○ Recieved treatment in the past<br>○ Currently on treatment		____yrs old	① ② ③ ④ ⑤ ⑥ ⑦ ⑧ ⑨<br>⓪ ① ② ③ ④ ⑤ ⑥ ⑦ ⑧ ⑨
Hepatitis C	○ Never received treatment<br>○ Recieved treatment in the past<br>○ Currently on treatment		____yrs old	① ② ③ ④ ⑤ ⑥ ⑦ ⑧ ⑨<br>⓪ ① ② ③ ④ ⑤ ⑥ ⑦ ⑧ ⑨
Liver cirrhosis	○ Never received treatment<br>○ Recieved treatment in the past<br>○ Currently on treatment	○ Yes<br>○ No	____yrs old	① ② ③ ④ ⑤ ⑥ ⑦ ⑧ ⑨<br>⓪ ① ② ③ ④ ⑤ ⑥ ⑦ ⑧ ⑨
Fatty liver	○ Never received treatment<br>○ Recieved treatment in the past<br>○ Currently on treatment		____yrs old	① ② ③ ④ ⑤ ⑥ ⑦ ⑧ ⑨<br>⓪ ① ② ③ ④ ⑤ ⑥ ⑦ ⑧ ⑨
Gastric ulcer / Duodenal ulcer	○ Never received treatment<br>○ Recieved treatment in the past<br>○ Currently on treatment	○ Yes<br>○ No	____yrs old	① ② ③ ④ ⑤ ⑥ ⑦ ⑧ ⑨<br>⓪ ① ② ③ ④ ⑤ ⑥ ⑦ ⑧ ⑨
Helicobacter Pylori bacterium	○ Never received treatment<br>○ Recieved treatment in the past<br>○ Currently on treatment		____yrs old	① ② ③ ④ ⑤ ⑥ ⑦ ⑧ ⑨<br>⓪ ① ② ③ ④ ⑤ ⑥ ⑦ ⑧ ⑨

Diagnosis	Treatment	Surgery	The time of diagnosis (age)	
Colon polyp	○ Never received treatment<br>○ Recieved treatment in the past<br>○ Currently on treatment	○ Yes<br>○ No	____yrs old	① ② ③ ④ ⑤ ⑥ ⑦ ⑧ ⑨<br>⓪ ① ② ③ ④ ⑤ ⑥ ⑦ ⑧ ⑨
Chronic Obstructive Pulmonary Disease	○ Never received treatment<br>○ Recieved treatment in the past<br>○ Currently on treatment		____yrs old	① ② ③ ④ ⑤ ⑥ ⑦ ⑧ ⑨<br>⓪ ① ② ③ ④ ⑤ ⑥ ⑦ ⑧ ⑨
Asthma	○ Never received treatment<br>○ Recieved treatment in the past<br>○ Currently on treatment		____yrs old	① ② ③ ④ ⑤ ⑥ ⑦ ⑧ ⑨<br>⓪ ① ② ③ ④ ⑤ ⑥ ⑦ ⑧ ⑨
Tuberculosis	○ Never received treatment<br>○ Recieved treatment in the past<br>○ Currently on treatment	○ Yes<br>○ No	____yrs old	① ② ③ ④ ⑤ ⑥ ⑦ ⑧ ⑨<br>⓪ ① ② ③ ④ ⑤ ⑥ ⑦ ⑧ ⑨
Benign breast tumor	○ Never received treatment<br>○ Recieved treatment in the past<br>○ Currently on treatment	○ Yes<br>○ No	____yrs old	① ② ③ ④ ⑤ ⑥ ⑦ ⑧ ⑨<br>⓪ ① ② ③ ④ ⑤ ⑥ ⑦ ⑧ ⑨
Hyperthyroidism or Hypothyroidism	○ Never received treatment<br>○ Recieved treatment in the past<br>○ Currently on treatment	○ Yes<br>○ No	____yrs old	① ② ③ ④ ⑤ ⑥ ⑦ ⑧ ⑨<br>⓪ ① ② ③ ④ ⑤ ⑥ ⑦ ⑧ ⑨
Benign thyroid tumor	○ Never received treatment<br>○ Recieved treatment in the past<br>○ Currently on treatment	○ Yes<br>○ No	____yrs old	① ② ③ ④ ⑤ ⑥ ⑦ ⑧ ⑨<br>⓪ ① ② ③ ④ ⑤ ⑥ ⑦ ⑧ ⑨
Kidney or bladder disease (including ureter stone)	○ Never received treatment<br>○ Recieved treatment in the past<br>○ Currently on treatment	○ Yes<br>○ No	____yrs old	① ② ③ ④ ⑤ ⑥ ⑦ ⑧ ⑨<br>⓪ ① ② ③ ④ ⑤ ⑥ ⑦ ⑧ ⑨
Benign prostatic hyperplasia	○ Never received treatment<br>○ Recieved treatment in the past<br>○ Currently on treatment	○ Yes<br>○ No	____yrs old	① ② ③ ④ ⑤ ⑥ ⑦ ⑧ ⑨<br>⓪ ① ② ③ ④ ⑤ ⑥ ⑦ ⑧ ⑨
Arthritis	○ Never received treatment<br>○ Recieved treatment in the past<br>○ Currently on treatment			① ② ③ ④ ⑤ ⑥ ⑦ ⑧ ⑨<br>⓪ ① ② ③ ④ ⑤ ⑥ ⑦ ⑧ ⑨
Cataract	○ Never received treatment<br>○ Recieved treatment in the past<br>○ Currently on treatment	○ Yes<br>○ No	____yrs old	① ② ③ ④ ⑤ ⑥ ⑦ ⑧ ⑨<br>⓪ ① ② ③ ④ ⑤ ⑥ ⑦ ⑧ ⑨
Glaucoma	○ Never received treatment<br>○ Recieved treatment in the past<br>○ Currently on treatment	○ Yes<br>○ No	____yrs old	① ② ③ ④ ⑤ ⑥ ⑦ ⑧ ⑨<br>⓪ ① ② ③ ④ ⑤ ⑥ ⑦ ⑧ ⑨
Herniated disc of spine	○ Never received treatment<br>○ Recieved treatment in the past<br>○ Currently on treatment	○ Yes<br>○ No	____yrs old	① ② ③ ④ ⑤ ⑥ ⑦ ⑧ ⑨<br>⓪ ① ② ③ ④ ⑤ ⑥ ⑦ ⑧ ⑨
Others ( )	○ Never received treatment<br>○ Recieved treatment in the past<br>○ Currently on treatment	○ Yes<br>○ No	____yrs old	① ② ③ ④ ⑤ ⑥ ⑦ ⑧ ⑨<br>⓪ ① ② ③ ④ ⑤ ⑥ ⑦ ⑧ ⑨

7. Are you taking any medications constantly more than a month? (including patch and inhalation medication)

○ No, I am not taking any of the following medication

If you have, please check the below.

Name of medicine	Treat or not	Name of medicine	Treat or not
Antihypertensive drug (Hypertension medication)	○ Currently on medication<br>○ Stopped medication	Thyroid disease medication	○ Currently on medication<br>○ Stopped medication
Antidiabetic medication (Oral medication or Insulin injection)	○ Currently on medication<br>○ Stopped medication	Osteoporosis medication	○ Currently on medication<br>○ Stopped medication
Lipid-lowering Agents (Dyslipidemia medication)	○ Currently on medication<br>○ Stopped medication	Hormone replacement therapy	○ Currently on medication<br>○ Stopped medication
Aspirin	○ Currently on medication<br>○ Stopped medication	Medication for BPH	○ Currently on medication<br>○ Stopped medication
Warfarin	○ Currently on medication<br>○ Stopped medication	Calcium	○ Currently on medication<br>○ Stopped medication
Other anticoagulant	○ Currently on medication<br>○ Stopped medication	Iron	○ Currently on medication<br>○ Stopped medication
Antiarrhythmic agent	○ Currently on medication<br>○ Stopped medication	Antidepressant, Sedative or other medications for psychiatric disease	○ Currently on medication<br>○ Stopped medication
Indigestion medication or Antacid	○ Currently on medication<br>○ Stopped medication	Respiratory agent	○ Currently on medication<br>○ Stopped medication
Hepatic drugs or Liver disease medication	○ Currently on medication<br>○ Stopped medication	Nutritional supplements	○ Currently on medication<br>○ Stopped medication
Constipation medication	○ Currently on medication<br>○ Stopped medication	Herbal, oriental medicine	○ Currently on medication<br>○ Stopped medication
Pain medication	○ Currently on medication<br>○ Stopped medication	Others	○ Currently on medication<br>○ Stopped medication

8. Have you ever had cancer?

○ No, I have never had any cancer.

If you have had cancer, please check the below.

Type of Cancer	Treatment		The time of diagnosis (age)	
○ Stomach cancer	○ Surgery<br>○ Radiation therapy	○ Chemotherapy<br>○ The others	____yrs old	① ② ③ ④ ⑤ ⑥ ⑦ ⑧ ⑨<br>⓪ ① ② ③ ④ ⑤ ⑥ ⑦ ⑧ ⑨
○ Lung cancer	○ Surgery<br>○ Radiation therapy	○ Chemotherapy<br>○ The others	____yrs old	① ② ③ ④ ⑤ ⑥ ⑦ ⑧ ⑨<br>⓪ ① ② ③ ④ ⑤ ⑥ ⑦ ⑧ ⑨
○ Liver cancer	○ Surgery<br>○ Radiation therapy	○ Chemotherapy<br>○ The others	____yrs old	① ② ③ ④ ⑤ ⑥ ⑦ ⑧ ⑨<br>⓪ ① ② ③ ④ ⑤ ⑥ ⑦ ⑧ ⑨
○ Colon cancer	○ Surgery<br>○ Radiation therapy	○ Chemotherapy<br>○ The others	____yrs old	① ② ③ ④ ⑤ ⑥ ⑦ ⑧ ⑨<br>⓪ ① ② ③ ④ ⑤ ⑥ ⑦ ⑧ ⑨
○ Breast cancer	○ Surgery<br>○ Radiation therapy	○ Chemotherapy<br>○ The others	____yrs old	① ② ③ ④ ⑤ ⑥ ⑦ ⑧ ⑨<br>⓪ ① ② ③ ④ ⑤ ⑥ ⑦ ⑧ ⑨
○ Cervical cancer	○ Surgery<br>○ Radiation therapy	○ Chemotherapy<br>○ The others	____yrs old	① ② ③ ④ ⑤ ⑥ ⑦ ⑧ ⑨<br>⓪ ① ② ③ ④ ⑤ ⑥ ⑦ ⑧ ⑨
○ Thyroid cancer	○ Surgery<br>○ Radiation therapy	○ Chemotherapy<br>○ The others	____yrs old	① ② ③ ④ ⑤ ⑥ ⑦ ⑧ ⑨<br>⓪ ① ② ③ ④ ⑤ ⑥ ⑦ ⑧ ⑨

Type of Cancer	Treatment		The time of diagnosis (age)	
○ Bladder cancer	○ Surgery<br>○ Radiation therapy	○ Chemotherapy<br>○ The others	____yrs old	① ② ③ ④ ⑤ ⑥ ⑦ ⑧ ⑨<br>⓪ ① ② ③ ④ ⑤ ⑥ ⑦ ⑧ ⑨
○ Esophagus cancer	○ Surgery<br>○ Radiation therapy	○ Chemotherapy<br>○ The others	____yrs old	① ② ③ ④ ⑤ ⑥ ⑦ ⑧ ⑨<br>⓪ ① ② ③ ④ ⑤ ⑥ ⑦ ⑧ ⑨
○ Gallbladder cancer	○ Surgery<br>○ Radiation therapy	○ Chemotherapy<br>○ The others	____yrs old	① ② ③ ④ ⑤ ⑥ ⑦ ⑧ ⑨<br>⓪ ① ② ③ ④ ⑤ ⑥ ⑦ ⑧ ⑨
○ Ovarian cancer	○ Surgery<br>○ Radiation therapy	○ Chemotherapy<br>○ The others	____yrs old	① ② ③ ④ ⑤ ⑥ ⑦ ⑧ ⑨<br>⓪ ① ② ③ ④ ⑤ ⑥ ⑦ ⑧ ⑨
○ Prostate cancer	○ Surgery<br>○ Radiation therapy	○ Chemotherapy<br>○ The others	____yrs old	① ② ③ ④ ⑤ ⑥ ⑦ ⑧ ⑨<br>⓪ ① ② ③ ④ ⑤ ⑥ ⑦ ⑧ ⑨
○ Pancreas cancer	○ Surgery<br>○ Radiation therapy	○ Chemotherapy<br>○ The others	____yrs old	① ② ③ ④ ⑤ ⑥ ⑦ ⑧ ⑨<br>⓪ ① ② ③ ④ ⑤ ⑥ ⑦ ⑧ ⑨
○ Others cancer<br>( )	○ Surgery<br>○ Radiation therapy	○ Chemotherapy<br>○ The others	____yrs old	① ② ③ ④ ⑤ ⑥ ⑦ ⑧ ⑨<br>⓪ ① ② ③ ④ ⑤ ⑥ ⑦ ⑧ ⑨

9. Have your family members ever been diagnosed with any of the followings?

○ No, my family members have never been diagnosed with any of the followings.

If you have, please check the below.

Diagnosis	Father	Mother	Siblings	Children	Paternal grandfather	Paternal grandmother	Maternal grandfather	Maternal grandmother
Hypertension	○	○	○	○	○	○	○	○
Diabetes	○	○	○	○	○	○	○	○
Myocardial infarction / Angina	○	○	○	○	○	○	○	○
Stroke	○	○	○	○	○	○	○	○
Dementia	○	○	○	○	○	○	○	○
Chronic hepatitis or Liver cirrohsis	○	○	○	○	○	○	○	○
Asthma or COPD	○	○	○	○	○	○	○	○
Stomach cancer	○	○	○	○	○	○	○	○
Lung cancer	○	○	○	○	○	○	○	○
Liver cancer	○	○	○	○	○	○	○	○
Colon cancer	○	○	○	○	○	○	○	○
Breast cancer	○	○	○	○	○	○	○	○
Cervical cancer	○	○	○	○	○	○	○	○
Thyroid cancer	○	○	○	○	○	○	○	○
Bladder cancer	○	○	○	○	○	○	○	○
Esophageal cancer	○	○	○	○	○	○	○	○
Gallbladder cancer	○	○	○	○	○	○	○	○
Ovarian cancer	○	○	○	○	○	○	○	○
Prostate cancer	○	○	○	○	○	○	○	○
Pancreas cancer	○	○	○	○	○	○	○	○
Others ( )	○	○	○	○	○	○	○	○

Classified By Body System

If you have any symptoms among the following, please check.

1. The digestive system

- ○ Difficulty swallowing
- ○ Having water brash or reflux
- ○ Frequent nausea or vomiting
- ○ Bloating, gas or indigestion
- ○ Heartburn
- ○ Black tarry stool
- ○ Stool with fresh blood
- ○ Frequent diarrhea
- ○ Constipation
- ○ Thin-sized stool, like a pencil
- ○ Palpable abdominal mass
- ○ Frequent stomachache

4. The Heart and Vascular system

- ○ Tightness and/or heavy pressure on chest with radiating pain to the arm, back or neck
- ○ Shortness of breath or chest discomfort on exertion
- ○ Hard, fast or irregular heartbeat
- ○ Swelling of face, ankles or legs
- ○ Chest discomfort or shortness of breath aggravated by lying down and relieved by sitting up.
- ○ Calf muscle pain during walking.

2. The respiratory system

- ○ Persistent cough
- ○ Yellowish or greenish sputum(phlegm)
- ○ Blood-tinged sputum
- ○ Wheezing sound when breathing
- ○ Shortness of breath

5. Metabolism and the endocrine system

- ○ Loss of appetite
- ○ Brittle nails and thinning hair
- ○ Frequent thirst
- ○ Frequent facial flushing
- ○ Sensitive to the cold
- ○ Sensitive to the heat

3. The kidney and urinary system

- ○ Amount of urine is getting increased
- ○ Voiding difficulty or residual urine sense
- ○ Difficult to postpone urination
- ○ Pain during urinating
- ○ Weakened stream
- ○ Red or coke-colored urine
- ○ Lower abdomen or flank pain
- ○ Wake at night for voiding
- ○ Incontinence of urine
- ○ Problem in having sexual intercourse

6. The nervous and mental system

- ○ Frequent dizziness
- ○ Headache
- ○ History of fainting
- ○ History of paralysis of extremities
- ○ Numb hands and feet
- ○ Slowness of movement or tremor
- ○ Forgetfulness, memory problems or poor concentration
- ○ Anxiety or depression
- ○ Whirling type dizziness

7. Musculoskeletal system

- ◯ Back pain
- ◯ Knee pain
- ◯ Shoulder pain
- ◯ Neck stiffness
- ◯ Joint soreness and stiffness
- ◯ History of swollen or painful joint
- ◯ Problems with joint movement

8. The dental system

- ◯ Toothache or mouth pain
- ◯ Bleeding gums
- ◯ Bad breath (Mouth odor)
- ◯ Dental scaling more than once per a year
- ◯ Dental implant
- ◯ Jaw pain

9. The others

- ◯ Involuntary weight loss (more than 10% during the last 6 months)
- ◯ Being tired easily
- ◯ Exhausted
- ◯ Fever or chill
- ◯ Problems with sleeping
- ◯ Easily bruising or nose bleeding
- ◯ Itchy skin
- ◯ Skin rash
- ◯ Get hives easily
- ◯ Recent blurred vision
- ◯ Sudden double vision
- ◯ Ringing in the ears
- ◯ Hoarseness over 2~3 weeks

Questions Only for Men

International Prostate Symptom Score

Question \ Score	not at all	less than 1 time in 5	less than half the time	about half the time	more than half the time	almost always
	0	1	2	3	4	5
**1. Incomplete Emptying** Over the past month, how often have you had a sensation of not emptying your bladder completely after you finish urinating?	○	○	○	○	○	○
**2. Frequency** Over the past month, how often have you had to urinate again less than every two hours?	○	○	○	○	○	○
**3. Intermittency** Over the past month, how often have you found you stopped and started again several times when you urinated?	○	○	○	○	○	○
**4. Urgency** Over the last month, how often have you found it difficult to postpone urination?	○	○	○	○	○	○
**5. Weak Stream** Over the past month, how often have you had a weak urinary stream?	○	○	○	○	○	○
**6. Straining** Over the past month, how often have you had to strain to start urination?	○	○	○	○	○	○
**7. Nocturia** Over the past month, how many times did you typically get up at night to urinate?	none	1	2	3	4	more than 5
	○	○	○	○	○	○

Quality of life due to urinary symptoms

Question \ Score	0	1	2	3	4	5	6
	○	○	○	○	○	○	○
If you were to spend the rest of your life with your urinary condition the way it is now, how would you feel about that?	Delighted	Pleased	Mostly satisfied	Mixed-about equally satisfied and dissatisfied	Mostly dissatisfied	Unhappy	Terrible

Questions Only for Women

1. When was your first period? (age)

under 10	11	12	13	14	15	16	17	18	19
○	○	○	○	○	○	○	○	○	○

2. Are you missing a period more than a year?

① Yes ② No ③ Irregularly ④ Same as usual

☞ **if your answer is ①, please answer the followings.**

2-1. At what age did it stop?

____year-old	① ② ③ ④ ⑤ ⑥ ⑦ ⑧ ⑨<br>⓪ ① ② ③ ④ ⑤ ⑥ ⑦ ⑧ ⑨

2-2. What is the reason?

① naturally because of the age
② after hysterectomy (surgical removal of uterus)
③ after oophorectomy (surgical removal of ovary)
④ after radiation therapy
⑤ after medication (ex.oral contraceptive)

3. If you have taken the female hormone treatment (pills, injection, patch, cream, vaginal tab, etc.), please answer the followings.

3-1. Total period for medical care?

① less than a year ② 1~3 years ③ 3~5 years
④ 5~10 years ⑤ more than 10 years

3-2. Are you still on the treatment?

① Yes ② No, quit

4. If you have ever been pregnant, please answer the followings.

4-1. Total number of delivery?

numbers	1	2	3	4	5	6	7	8	9	over 10
Total ______	○	○	○	○	○	○	○	○	○	○

4-2. Which method did you use for delivery?

numbers	1	2	3	4	5	6	7	8	9	over 10
Natural childbirth (Vaginal delivery) ____________	○	○	○	○	○	○	○	○	○	○

numbers	1	2	3	4	5	6	7	8	9	over 10
Caesarean operation ____________	○	○	○	○	○	○	○	○	○	○

4-3. Have you had an abortion?

① Yes ② No

☞ 4-3-1. If yes, how many times?

numbers	1	2	3	4	5	6	7	8	9	over 10
Spontaneous (miscarriage) __________	○	○	○	○	○	○	○	○	○	○

numbers	1	2	3	4	5	6	7	8	9	over 10
Induced abortion ______	○	○	○	○	○	○	○	○	○	○

4-4. Have you ever given a premature birth?

① Yes ② No

☞ 4-3-1. If yes, how many times?

numbers	1	2	3	4	5	6	7	8	9	over 10
Total _______	○	○	○	○	○	○	○	○	○	○

Stress Questionnaire

※ **Check the response which best indicates how often you experience each stress indicator during last few weeks.**

Items	always	mostly	some-times	not at all
1. Feel very healthy and comfortable	○	○	○	○
2. Do not feel refreshed right after the sleep	○	○	○	○
3. Being totally exhausted	○	○	○	○
4. Difficult to have a sound sleep because of the anxieties	○	○	○	○
5. Healthy mind	○	○	○	○
6. Feel full of energy	○	○	○	○
7. Being disturbed in mind or being nervous at night	○	○	○	○
8. Be good at self-managing like others	○	○	○	○
9. Feel everything is alright what I am doing.	○	○	○	○
10. Satisfied with the process or ways that I've done	○	○	○	○
11. Do not hesitate to start something	○	○	○	○
12. Can normally enjoy the daily life	○	○	○	○
13. Being irritated or being mean	○	○	○	○
14. Can solve the problems which are just around the corner	○	○	○	○
15. Feel unhappy and become depressed	○	○	○	○
16. Decreasing trusty about myself	○	○	○	○
17. Feel happy according to everything around me	○	○	○	○
18. Feel that my life is worth living	○	○	○	○

1. Do you feel stressed in your work?

① Strongly disagree ② Disagree ③ Neither agree or disagree
④ Agree ⑤ Strongly Agree

2. Do you feel worried or anxious with family relationship? (with spouse, children, and others)

① Strongly disagree ② Disagree ③ Neither agree or disagree
④ Agree ⑤ Strongly Agree

3. Do you need to take consultation for managing the stress?

① Yes ② No
