# Supplementary material for: Metabolically-healthy obesity is associated with higher prevalence of colorectal adenoma
Source: PLoS One. 2017 Jun 21;12(6):e0179480. doi: 10.1371/journal.pone.0179480 (PMC5479542; doi:10.1371/journal.pone.0179480)
Supplement: S1 Table — (DOCX) [file pone.0179480.s003.docx]

**S1 Table. Association between body mass index and colorectal adenoma.**

| BMI (per unit) | Unadjusted | Model 1 | Model 2 | Model 3 |
| --- | --- | --- | --- | --- |
| Overall |  |  |  |  |
| Any adenoma | 1.09 (1.08-1.10) | 1.05 (1.04-1.06) | 1.05 (1.04-1.06) | 1.03 (1.02-1.05) |
| Multiple adenoma^a^ | 1.12 (1.11-1.14) | 1.07 (1.05-1.08) | 1.07 (1.06-1.09) | 1.06 (1.04-1.08) |
| High-risk adenom^a^ | 1.12 (1.10-1.14) | 1.07 (1.05-1.09) | 1.07 (1.05-1.09) | 1.06 (1.04-1.08) |
| Metabolically-healthy |  |  |  |  |
| Any adenoma | 1.10 (1.07-1.12) | 1.04 (1.02-1.06) | 1.04 (1.02-1.06) | 1.04 (1.02-1.07) |
| Multiple adenoma^a^ | 1.17 (1.14-1.21) | 1.09 (1.06-1.13) | 1.10 (1.06-1.13) | 1.10 (1.06-1.14) |
| High-risk adenoma^a^ | 1.14 (1.10-1.19) | 1.07 (1.02-1.11) | 1.07 (1.03-1.12) | 1.08 (1.03-1.13) |
| Metabolically-unhealthy |  |  |  |  |
| Any adenoma | 1.06 (1.05-1.08) | 1.04 (1.03-1.05) | 1.05 (1.03-1.06) | 1.03 (1.02-1.05) |
| Multiple adenoma^a^ | 1.08 (1.06-1.10) | 1.06 (1.04-1.08) | 1.07 (1.05-1.09) | 1.05 (1.03-1.07) |
| High-risk adenom^a^ | 1.08 (1.06-1.11) | 1.07 (1.04-1.09) | 1.07 (1.05-1.10) | 1.06 (1.03-1.09) |

Values in parenthesis are 95% confidence intervals. ^a^ Compared to individuals without adenoma. Abbreviations: BMI, body mass index. Model 1: Adjusted for age and sex. Model 2: Further adjusted for smoking, alcohol, first-degree family history of colorectal cancer, and aspirin use. Model 3: Further adjusted for fasting blood glucose, systolic blood pressure, triglyceride, high-density lipoprotein cholesterol, low-density lipoprotein cholesterol, and HOMA-IR.
