# Supplementary material for: Metabolically-healthy obesity is associated with higher prevalence of colorectal adenoma
Source: PLoS One. 2017 Jun 21;12(6):e0179480. doi: 10.1371/journal.pone.0179480 (PMC5479542; doi:10.1371/journal.pone.0179480)
Supplement: S2 Table — (DOCX) [file pone.0179480.s004.docx]

**S2 Table. Prevalence and odd ratios of colorectal adenoma by body mass index category among metabolically-healthy men and women.**

|  | Men (n = 3,630) | | | | Women (n = 5,552) | | | |
| --- | --- | --- | --- | --- | --- | --- | --- | --- |
|  | Underweight (n = 52) | Normal (n = 1,421) | Overweight (n = 1,191) | Obese (n = 996) | Underweight (n = 321) | Normal (n = 3,778) | Overweight (n = 1,004) | Obese (n = 449) |
| Any adenoma |  |  |  |  |  |  |  |  |
| Prevalence, % | 32.7 | 34.1 | 34.0 | 37.2 | 18.1 | 18.6 | 21.1 | 25.2 |
| Unadjusted | 0.94 (0.52-1.69) | reference | 0.99 (0.84-1.17) | 1.14 (0.96-1.35) | 0.96 (0.71-1.29) | reference | 1.17 (0.98-1.39) | 1.47 (1.17-1.84) |
| Adjusted |  |  |  |  |  |  |  |  |
| Model 1 | 0.79 (0.43-1.45) | reference | 0.99 (0.84-1.17) | 1.17 (0.98-1.39) | 1.00 (0.74-1.35) | reference | 1.08 (0.90-1.28) | 1.33 (1.06-1.68) |
| Model 2 | 0.75 (0.41-1.39) | reference | 0.99 (0.84-1.17) | 1.20 (1.00-1.42) | 0.99 (0.73-1.34) | reference | 1.07 (0.90-1.28) | 1.35 (1.07-1.70) |
| Model 3 | 0.78 (0.42-1.44) | reference | 0.97 (0.82-1.16) | 1.16 (0.96-1.40) | 0.99 (0.73-1.34) | reference | 1.08 (0.90-1.29) | 1.36 (1.07-1.73) |
| Multiple adenomas^a^ |  |  |  |  |  |  |  |  |
| Prevalence, % | 11.5 | 12.9 | 13.7 | 15.2 | 2.5 | 3.8 | 6.8 | 9.4 |
| Unadjusted | 0.87 (0.36-2.11) | reference | 1.06 (0.84-1.33) | 1.24 (0.97-1.57) | 0.65 (0.31-1.33) | reference | 1.83 (1.36-2.47) | 2.66 (1.85-3.83) |
| Adjusted |  |  |  |  |  |  |  |  |
| Model 1 | 0.63 (0.25-1.59) | reference | 1.08 (0.85-1.37) | 1.34 (1.05-1.71) | 0.69 (0.33-1.43) | reference | 1.55 (1.14-2.10) | 2.19 (1.51-3.18) |
| Model 2 | 0.59 (0.23-1.39) | reference | 1.09 (0.86-1.39) | 1.39 (1.08-1.79) | 0.68 (0.32-1.41) | reference | 1.55 (1.14-2.11) | 2.25 (1.55-3.27) |
| Model 3 | 0.59 (0.23-1.51) | reference | 1.09 (0.85-1.39) | 1.39 (1.07-1.82) | 0.70 (0.33-1.46) | reference | 1.53 (1.12-2.10) | 2.25 (1.52-3.32) |
| High-risk adenoma^a^ |  |  |  |  |  |  |  |  |
| Prevalence, % | 5.8 | 7.3 | 6.4 | 7.8 | 2.5 | 2.1 | 3.2 | 5.1 |
| Unadjusted | 0.77 (0.23-2.55) | reference | 0.87 (0.63-1.18) | 1.11 (0.81-1.52) | 1.16 (0.55-2.44) | reference | 1.55 (1.02-2.35) | 2.63 (1.63-4.24) |
| Adjusted |  |  |  |  |  |  |  |  |
| Model 1 | 0.48 (0.13-1.72) | reference | 0.90 (0.65-1.24) | 1.23 (0.89-1.70) | 1.24 (0.59-2.61) | reference | 1.34 (0.88-2.05) | 2.22 (1.37-3.61) |
| Model 2 | 0.45 (0.12-1.59) | reference | 0.92 (0.66-1.27) | 1.30 (0.94-1.81) | 1.24 (0.59-2.62) | reference | 1.32 (0.86-2.02) | 2.28 (1.40-3.73) |
| Model 3 | 0.47 (0.13-1.66) | reference | 0.90 (0.64-1.25) | 1.26 (0.89-1.79) | 1.22 (0.57-2.59) | reference | 1.32 (0.86-2.03) | 2.34 (1.40-3.90) |

Values in parenthesis are 95% confidence intervals. ^a^ Compared to individuals without adenoma. Model 1: Adjusted for age. Model 2: Further adjusted for smoking, alcohol, first-degree family history of colorectal cancer, and aspirin use. Model 3: Further adjusted for fasting blood glucose, systolic blood pressure, triglyceride, high-density lipoprotein cholesterol, low-density lipoprotein cholesterol, and HOMA-IR.
